# MnO_2_ efficiently removes indigo carmine dyes from polluted water

**DOI:** 10.1016/j.heliyon.2018.e00897

**Published:** 2018-11-07

**Authors:** K.P. Vidya Lekshmi, Suguna Yesodharan, E.P. Yesodharan

**Affiliations:** School of Environmental Studies, Cochin University of Science and Technology, Kochi 682022, India

**Keywords:** Environmental science, Inorganic chemistry, Physical chemistry

## Abstract

MnO_2_ is identified as a highly efficient sonocatalyst and sonophotocatalyst for the complete removal of even very small concentration of Indigo carmine (IC) dye pollutant from water. The effect of various reaction parameters, viz. dosage of the catalyst, concentration of pollutant, volume of reaction system, pH, dissolved gases, presence of anions/salts and oxidants etc. on the rate of degradation is evaluated and optimum parameters are identified. The degradation follows variable kinetics depending on the concentration of the substrate. The rate of degradation is facilitated by acidic pH. Classic oxidants H_2_O_2_ and S_2_O_8_^2−^ behave differently, with the former inhibiting and the latter enhancing the degradation. The effect of anions/salts on the degradation is complex and ranges from ‘inhibition’ (PO_4_^3−^, CO_3_^2−^, HCO_3_^−^) and ‘no effect’ (SO_4_^2−^, Cl^−^) to ‘enhancement’ (NO_3_^−^, CH_3_COO^−^). The high affinity of MnO_2_ for O_2_ and its extremely efficient adsorption of H_2_O_2_ and the substrate play key roles in the efficiency of the process. Participation of lattice oxygen from MnO_2_ in the reaction, whenever the dissolved or adsorbed oxygen is deficient, is an important highlight of the process. Major transient intermediates formed during the process are identified by LC–MS. Combination of sonocatalysis with UV photolysis (sonophotocatalysis) enhances the efficiency of degradation and mineralization of IC.

## 1 Introduction

Ultrasound (US) irradiation (sonication) and US – initiated heterogeneous catalysis (sonocatalysis) have been investigated extensively in recent years as potential tertiary treatment processes for the removal of even very small concentration of chemical and bacterial pollutants from water [1, 2, 3, 4, 5, 6, 7, 8, 9]. These advanced oxidation processes (AOP) have been proven effective for the degradation and eventual mineralization of many types of recalcitrant organic pollutants such as pesticides, phenols, pharmaceuticals and dyes. Sonocatalysis mediated by zinc oxide has been demonstrated as highly efficient for the complete and irreversible destruction of bacterial pollutants such as *Escherichia coli, Bacillus subtilis, Vibrio harveyi* and *Pseudomonas aeruginosa* [4, 6]. However, US based processes are generally less efficient for the removal of chemical contaminants compared to other similar AOPs such as photocatalysis or electrocatalysis under identical reaction conditions [7, 8, 9]. Attempts to enhance the efficiency of sonocatalysis for wastewater treatment are mostly confined to their combination with additives such as H_2_O_2_, H_2_O_2_/Fe^2+^, Fe^3+^, cations, anions, dyes etc. and hybrid energy sources including light and microwave. Another approach is to modify the characteristics of the catalyst by doping, supporting and coating [10, 11, 12, 13, 14].

### 1.1 Theory

Ultrasound waves in liquid create the phenomenon of ‘cavitation’ which involves the formation, growth and eventual collapse of microbubbles. This results in the release of large quantum of energy thereby inducing extreme local conditions of temperature and pressure. Sonochemical destruction of pollutants in water involves multiple reaction pathways including pyrolysis inside the liquid bubble and hydroxyl radical-mediated reactions at the bubble-liquid interface and/or in the liquid bulk.

A bubble in a liquid is inherently unstable. On irradiation with ultrasound it absorbs energy from alternating compression and expansion cycles of the sound wave [15]. Consequently the bubbles grow and contract, eventually striking a dynamic balance between the vapor inside the bubble and the liquid outside. The growth of the cavity depends on the intensity of sound. Under high intensity ultrasound, cavities grow rapidly while under low intensity sound they grow very slowly over many cycles. The growing cavity can eventually reach a critical size (which depends on the frequency of the ultrasound wave) where it will absorb energy efficiently from the ultrasound. At this stage, the cavity grows rapidly and concurrently the efficiency of energy absorption from ultrasound declines. The cavity can no longer sustain without the energy input and consequently the liquid rushes in and the cavity implodes. The intensity of cavity implosion depends on acoustic frequency and intensity, ambient temperature, static pressure, characteristics of the liquid and ambient gas etc. The bubble collapse results in localized supercritical condition such as high temperature, pressure, electrical discharges and plasma effects [1, 2, 3, 16, 17]. The gaseous contents of the collapsing cavity can reach temperatures of the order of ∼5500 °C. Temperature of the liquid immediately surrounding the cavity can be as high as ∼ 2100 °C. The localized pressure can reach as high as ∼500 atmospheres. These conditions, which are explained based on the ‘hot spot theory’ result in the formation of transient supercritical water [18]. Consequently each microbubble can act as a small microreactor which produces different reactive species and heat during its collapse [19].

Under ultrasound irradiation sonolysis of water and thermal dissociation of O_2_ will produce highly reactive species such as ^·^OH, H^·^, O^·^ and HO_2_^·^. Sonolysis of water can also produce H_2_O_2_ and H_2_ via ^·^OH and H^·^. This highly reactive environment with multitude of reactive free radicals is congenial for the decomposition and eventual mineralization of organic pollutants.

Besides chemical effects, ultrasound can also produce physical effects (sonophysical). Liquid medium will absorb the acoustic energy from sound waves and flow along the wave's propagation direction. Physical effects such as microstreaming, microjets and shock waves can also be produced by cavitation bubbles. This results in turbulent fluid movement and a microscale velocity gradient in the vicinity of cavitation bubbles [20, 21]. Other effects of US such as increase in the surface area of suspended particles due to fragmentation as well as deagglomeration, continuous cleaning of the surface by microstreaming etc. can be used beneficially in sonocatalysis. This will help in the sustained efficiency of the catalyst for repeated application.

Simple sonolysis of aqueous systems is not efficient enough to achieve the mineralization of many chemical pollutants in water [3, 4]. However, in presence of appropriate catalysts, the US effects of cavitation and consequent processes are significantly enhanced resulting in faster degradation and mineralization of many types of pollutants. US irradiation produces dramatic changes in surface morphology of solids [15]. Particle surfaces get smoothened and polished. Particles can get deaggregated or sometimes even consolidated as extended aggregates. Further, US induced fluid movement can enhance the physical mass transfer processes between solid–liquid and liquid–gas interfaces resulting in better mixing, breaking down of particles, degradation and desorption of molecules, cleaning of surfaces etc. When a cavitation bubble collapses asymmetrically near a micro particle surface, high velocity microjets are formed and together with the asymmetric shock wave generated upon implosion of the bubbles, the particle surface is eroded and cleaned. These factors will lead to enhanced effects by heterogeneous catalysts in solid–liquid systems. Due to constant clean-up, desorption of the surface and deaggregation of particles, the catalytic activity can be enhanced manifold and sustained over longer periods. This factor can be used beneficially for the removal of recalcitrant chemical pollutants from water using sonocatalysis.

### 1.2 Typical commercially available sonocatalysts

Almost all reported sonocatalytic studies on water purification use SiO_2_, ZnO and TiO_2_ as catalysts probably because of better availability, convenience, low cost and the possibility of exploiting the photocatalytic potential resulting from the sonoluminescence [3, 4, 5, 22, 23]. Nakajima et al. [23] reported that TiO_2_ is more efficient than SiO_2_ for the sonochemical destruction of 1,4-dioxane in aqueous systems. Dadjour et al. [24] reported that TiO_2_ is more effective than Al_2_O_3_ for disinfection of *E coli* while Anju et al. [4] and Vidya Lekshmi et al. [6] reported the high activity of ZnO for the sonocatalytic removal of a variety of gram positive and gram negative organisms. The relatively higher sonocatalytic activity of photosensitive semiconductor oxides such as ZnO and TiO_2_ has been attributed to the combination of sonocatalysis and sonication-induced photocatalysis initiated by the intense UV light of single bubble sonoluminescence [5, 25]. Keck et al. [17] attributes the enhanced degradation in presence of even catalytically inactive particles to the change in the shape of US induced bubbles from spherical to asymmetric. The large surface area of these bubbles enable more insitu formed reactive radicals to escape into the bulk and interact with the substrate.

### 1.3 MnO_2_ as AOP catalyst

In the present work, a classic heterogeneous catalyst MnO_2_ is investigated for its potential as a sonocatalyst for the removal of a recalcitrant dye pollutant Indigo carmine (IC) from water. Only very few investigations aimed at the complete removal of very small concentration of IC from water have been reported and these are mostly based on Advanced Oxidation Processes (AOP), especially photocatalysis and microwave catalysis [26, 27, 28, 29, 30].

The characteristics of MnO_2_ useful in catalysis are its excellent adsorption and oxidation properties. The oxidation-reduction chemistry of MnO_2_ is especially important in electron-transfer reactions due to the presence of Mn ions in different oxidation states. MnO_2_ is known to be a good catalyst for water splitting reactions [31]. It has also been proven to be a good catalyst for splitting H_2_O_2_ to form reactive free radicals such as ^·^OH and HO_2_^·^ [32]. Hence the insitu formed H_2_O_2_ in AOPs can be beneficially transformed to highly reactive oxygen species (ROS) such as ^·^OH and HO_2_^·^ in presence of MnO_2_. Manganese is known to be a necessary element in many biological systems and hence MnO_2_ will be an environmentally safer catalyst to be used in water treatment. Of late, various types of MnO_2_ based catalysts have been subjected to extensive investigations to explore their potential for water purification. Liu et al. [33] synthesized highly porous MnO_2_ with different particle sizes using a one-pot hydration and annealing process and their catalytic performances were evaluated under different conditions using the degradation of phenol in aqueous solutions as the test reaction. The smaller particle size of the catalyst and the annealing treatments improved the catalytic activity and stability significantly. Yang et al. [34] investigated the catalytic behaviors of Oxone-MnO_x_/silica (MS) systems towards aqueous ibuprofen (IBU) degradation using various parameters and demonstrated the strong influence of pH on the degradation rate. The catalytic efficacy of MS was inhibited by various anions and humic acid to different extents. Modification of γ- MnO_2_ by incorporating Fe into MnO_2_ hollow microsphere framework resulted in enhanced performance for As(III) removal compared to the undoped counterparts [35]. The enhancement is attributed to the unique structural characteristics of highly accessible surface and fully exposed active sites inherited from the hierarchical iron-containing γ-MnO_2_ samples. Concurrent adsorption and oxidation, in which MnO_2_ oxidises the As(III) to As(V) and ferrous species adsorbs the generated As(V), also contribute to the enhancement. Another innovation is the ultrasonic atomization-assisted synthesis of self-assembled MnO_2_ octahedral molecular sieve nanostructures and their application as catalysts in water treatment [36]. In the case of MnO_*x*_/SBA-15 (MS) composites synthesized under different conditions, the physicochemical features and catalytic efficacies depend on the Mn/SBA-15 weight ratio, calcination temperature, specific surface area and the types/amounts of MnO_*x*_ species of the composites [37]. The types and the amounts of reactive free radicals also depend on the conditions of synthesis.

### 1.4 Current limitations of AOP

All advanced oxidation processes including sonocatalysis, photocatalysis and sonophotocatalysis have limitations at the current level of knowledge, especially with respect to economical commercial application, in spite of the growing number of publications on newer applications, improved catalysts and reaction mechanisms. There is wide disconnect between research directions and the actual need of the society/industry [38]. The energy-efficiency of the process needs to be improved substantially. The potential of relatively inexpensive environment-friendly, energy-efficient additives to enhance the viability of the process is not fully explored. The possibility of utilizing natural contaminants such as oxy anions (NO_3_^−^, SO_4_^2−^ etc.) in water as efficiency enhancers also needs in-depth investigation. More effective means of using simple inexpensive commercially available catalysts as well as the development of novel nano and composite catalysts and successful scale up of laboratory-level technologies also are important. Innovative reactor design as well as catalyst recovery and recycling techniques are other issues to be addressed. Ultimately, the cost-effectiveness of the AOP needs to exceed that of the existing technologies such as those based on peroxide and ozone, in order to make it commercially viable.

To the best of our knowledge, the current study is probably the first instance of the application of MnO_2_ mediated sonocatalysis for the degradation/mineralization of Indigo carmine dye in water.

## 2 Materials and methods

### 2.1 Materials

MnO_2_ (>99% purity) used in the study was from Merck India Limited. The particles were approximately spherical and nonporous. The surface area as determined by the BET method is ∼31 m^2^/g. The pore volume was ∼0.05 cm^3^/g and the average pore width was 74.2 Å. The particle size was analysed using Malvern Mastersizer 3000. The average particle size was 3.2 μm.

**Fig. 1:**
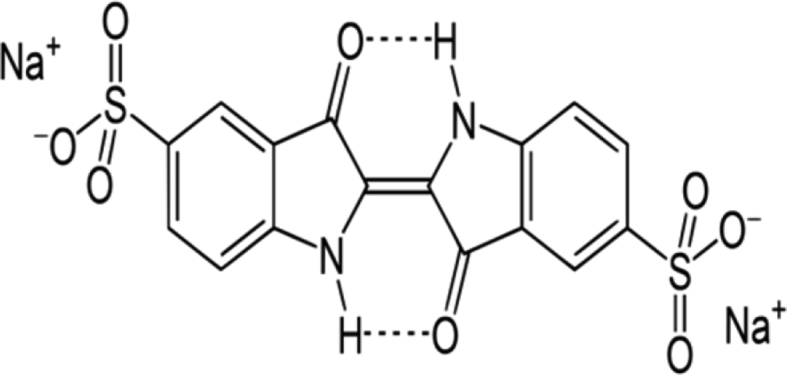
Structure of indigo carmine.

Indigo carmine is a toxic, potentially carcinogenic dye which can lead to reproductive, developmental, neuro and acute toxicity. Other health hazards include cardiovascular and respiratory effects, gastrointestinal irritation and possible tumors at the site of application [39]. The structure of IC is shown in Fig. 1.

Indigo Carmine (Extra Pure Grade >99.5% purity) from Sisco Research Laboratories Pvt. Ltd (India), was used as such without further purification. Sodium persulphate (PS) (purity ∼99%) and H_2_O_2_ (30.0% w/v) were from Qualigen (India) Limited. Doubly distilled water was used in all the experiments. All other chemicals were of AnalaR Grade or equivalent.

### 2.2 Instrumentation

X-ray diffraction measurement was made by using Rigaku X-ray diffractometer with CuKά radiation. Scanning Electron Microscopy (SEM) measurements were performed with JEOL Model JSM-6390 LV. Diffuse Reflectance Spectra (DRS) were recorded with Varian Cary 5000 using BaSO_4_ as the reference. Transmission electron microscopy (TEM) was done using JOEL/JEM/200; source LaB6. FTIR spectra were recorded using Nicolet Avatar 370 instrument equipped with triglycine sulfate detector with a KBr window and a KBr beam splitter. The equipment was purged with dry air to prevent interference from atmospheric moisture. The transmission spectrum was obtained with a resolution of 4 cm^−1^ by using 32 scans in the range 400–4000 cm^−1^. The powdered samples were diluted with finely ground KBr to ∼5% by weight.

### 2.3 Methodology

The sonocatalytic experiments were performed using aqueous solutions of IC of the desired concentration. Specified quantity of the catalyst was suspended in the dye solution. Experiments with and without mechanical mixing did not show significant difference in the sonocatalytic degradation of the dye thereby suggesting that sonication was sufficient to ensure adequate mixing of the suspension. However, mechanical mixing can influence the rate of degradation under sonication depending on the characteristics of the catalyst, substrate and sonication conditions [40]. In the current instance, the catalyst remains well suspended and is uniformly distributed in the reaction medium for longer time by sonication even without mechanical mixing.

A cylindrical Pyrex vessel of 250 ml capacity was used as the reactor. An ultrasonic bath (53/42 kHz, 100 W) was used as the source of US. Water from the sonicator was continuously replaced by circulation from a thermostat maintained at the required temperature. The reaction temperature was maintained at 29 ± 1 °C unless mentioned otherwise. The position of the reactor in the ultrasonic bath was always kept the same. Samples were drawn at periodic intervals, the suspended catalyst particles were removed by centrifugation and the concentration of IC left behind was analyzed by Spectrophotometry at 608 nm [27]. Reaction system kept in the dark under exactly identical conditions (without US irradiation) served as the reference. H_2_O_2_ in the system was determined by standard iodometry [41]. Mineralization of IC was tested by determining chemical oxygen demand (COD) using standard K_2_Cr_2_O_7_ oxidation method [42] and total organic carbon (TOC) measurements. TOC was determined using TOC analyser model Elementar analysensysteme GmbH.

The calorimetric power which is the precise measure of the energy conditions inside the reactor is calculated by the following equation [43]:

(1) Power (W) = (dT/dt) × C_p_ × M

where (dT/dt) is the increase in temperature per second, C_p_ is the heat capacity of water (4.2 J g^−1^) and M is the mass of water (g).

Adsorption studies were performed as follows [44]:

The catalyst (0.1 g) was suspended in 100 ml of IC solution in a 250 ml reaction flask. The pH was adjusted at the desired value. The suspension was stirred continuously for 2 hours at constant temperature of 29 ± 1 °C to achieve equilibrium and then kept undisturbed for another 2 hours. Thereafter, it was centrifuged at 3000 rpm for 10 minutes and the concentration of IC in the supernatant was determined colorimetrically. Keeping the suspension for longer time before centrifuging, with or without stirring did not make any significant difference in the results. Hence the 2 hour period is sufficient to ensure maximum adsorption. The adsorption of IC was calculated from the following equation;

(2) q_e_ = (C_0_ − C_e_)V/W

where C_0_ is the initial concentration of the adsorbate IC (mg/L), C_e_ is the equilibrium adsorbate concentration in solution (mg/L), V is the volume (in liter) of the solution, W is the mass (in gram) of the adsorbent MnO_2_ and q_e_ is the amount of IC adsorbed (mg per gram of the adsorbent).

The formation of ^·^OH radicals during the ultrasonic irradiation of the MnO_2_ reaction system was tested by the photoluminescence (PL) technique using terephthalic acid (TPA) as the probe molecule [45]. The hydroxyl radicals formed insitu react with TPA and form 2- hydroxy terephthalic acid (HTPA), which is a fluorescent molecule. The intensity of its PL is proportional to the concentration of ^·^OH radicals in the system. In this method, MnO_2_ at the standard experimental concentrations is suspended in aqueous solution of NaOH (2 × 10^−3^M) and TPA (2 × 10^−4^M) and irradiated by US. The PL spectrum of the product HTPA is recorded after every 5 minutes of irradiation in the range of 400–450 nm. The excitation wavelength was 315 nm. The PL intensity at 425 nm corresponds to the concentration of HTPA and hence indirectly of the ^·^OH radicals formed in the system. Shimadzu model RF-5301PC fluorescence spectrophotometer is used for recording the spectrum.

## 3 Results and discussion

**Fig. 2:**
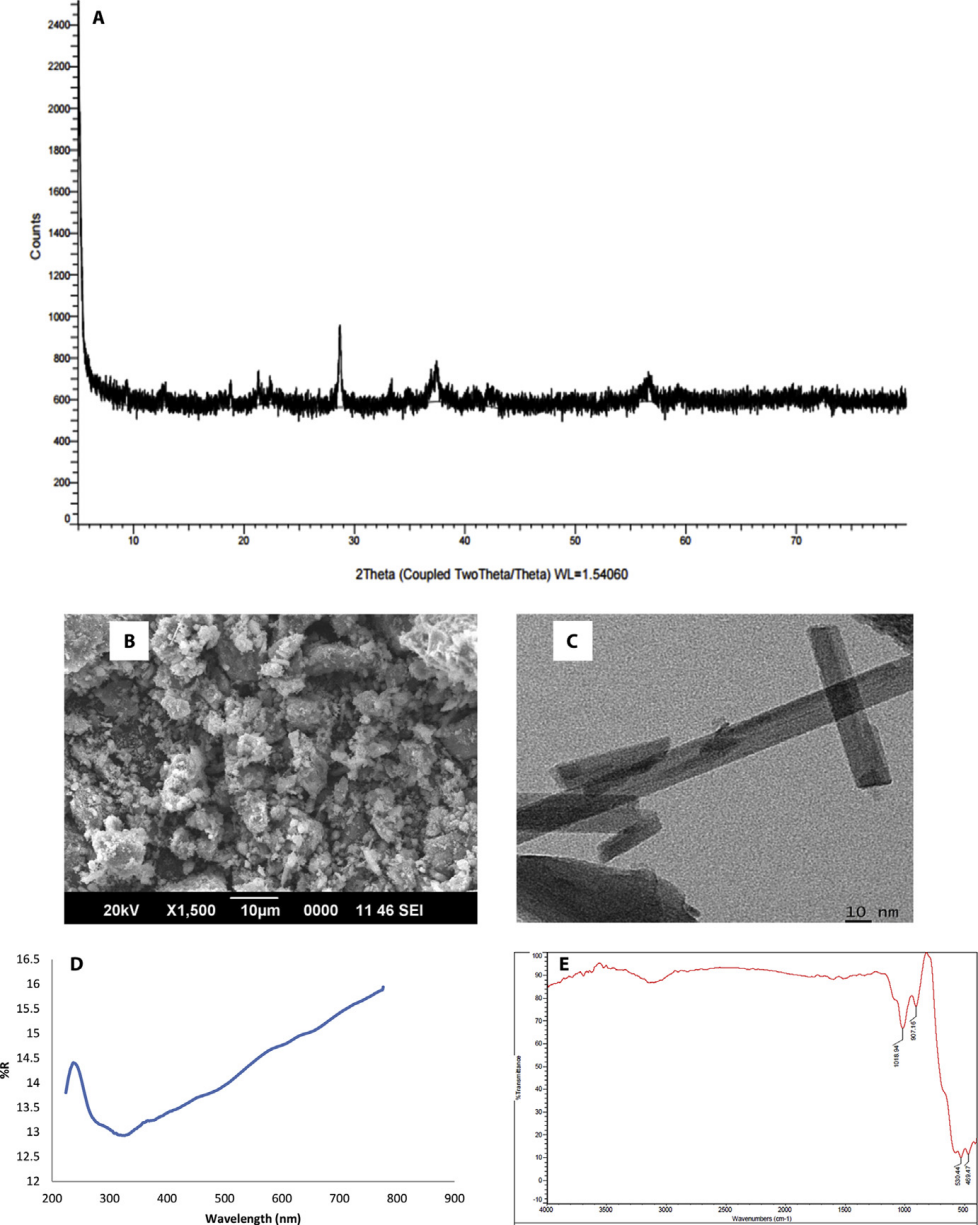
A: XRD of MnO_2_, B: SEM image and C: TEM image of MnO_2_, D: DRS of MnO_2_, E: FTIR spectrum of MnO_2_.

The catalyst MnO_2_ was characterized by X-ray diffraction (XRD), scanning electron microscopy (SEM), transmission electron microscopy (TEM), fourier transform infrared (FTIR) spectroscopy and diffuse reflectance spectroscopy (DRS). The XRD results show that the MnO_2_ is amorphous in nature with weak diffraction pattern (Fig. 2A). The morphology and particle size analysis were done using SEM (Fig. 2B). Particles were approximately rod-shaped with average particle size in the range of 220 nm. TEM analysis (Fig. 2C) also showed platelet and rod like structure. DRS (Fig. 2D) shows sharp reflectance band between 200 and 300 nm, indicating that the material absorbs in the UV range. The optical absorption intensity of MnO_2_ steadily increases from 300 to 800 nm with no clear band edge. This indicates that MnO_2_ is not a typical semiconductor like TiO_2_ or ZnO. The brown color of MnO_2_ also indicates favorable absorption of visible light [32]. The FTIR spectrum (Fig. 2E) shows very week absorption intensity of OH (3100–3600 cm^−1^) or H_2_O (1600 and 3600 cm^−1^). The general spectral pattern shows that MnO_2_ is pure with no contamination from any of the precursors.

All ultrasound experiments described here are done at constant sonicator power of 100 W. The difference in temperature of water in the sonicator and the solution inside the reactor at different times of irradiation was <1 °C. Hence the energy conditions inside as well as outside (in the sonicator) of the reactor are not much different. The calorimetric power which is the true measure of the energy inside the reactor under the reaction conditions is computed as 5.6 W mL^−1^ (unless indicated otherwise) as described in Section 2.3.

Preliminary investigations on the decolorization/degradation of IC in the presence of MnO_2_ and US individually and in combination under identical conditions showed that both the catalyst and US are essential for significant degradation. Very small quantity of IC degraded under US irradiation in the absence of the catalyst. Water is known to dissociate under US irradiation into reactive free radicals H^·^ and ^·^OH (reaction 3).

(3) $${\text{H}}_{2}\text{O}\overset{\text{>>>}}{\to }{\text{H}}^{\cdot }{+}^{\cdot }\text{OH}$$

These radicals are capable of attacking the organic compounds in solution [46] resulting in their degradation and possible mineralization. In addition to the ^·^OH radical-mediated degradation, pyrolytic degradation and oxidation by insitu formed H_2_O_2_ also take place, though only to limited extent [47]. Assuming pseudo first order kinetics as is the case with most AOPs, the rate constants for the sonolytic degradation of IC in the absence as well as presence of MnO_2_ are calculated. The presence of suspended MnO_2_ enhances the degradation significantly, at least by three times, in the present context. There is moderate reduction in the concentration of IC in presence of MnO_2_ even without US irradiation, i.e. ∼ 10% as against ∼45% with irradiation, in 2 minutes. This is due to simple adsorption. This also shows that more than 75% of the removal of IC in the simultaneous presence of MnO_2_ and US is due to degradation of the dye and not simple adsorption. The adsorption of IC at different concentrations on MnO_2_ is measured. At constant weight of MnO_2_ (1800 mg/L) the adsorption increases with increase in concentration of the dye (10–70 mg/L) and reaches a steady adsorption of ∼15 mg/L at IC concentration of 60 mg/L. The effect of various reaction parameters on the MnO_2_ mediated sonocatalytic degradation of IC is investigated in detail as follows:

### 3.1 Effect of catalyst dosage

**Fig. 3:**
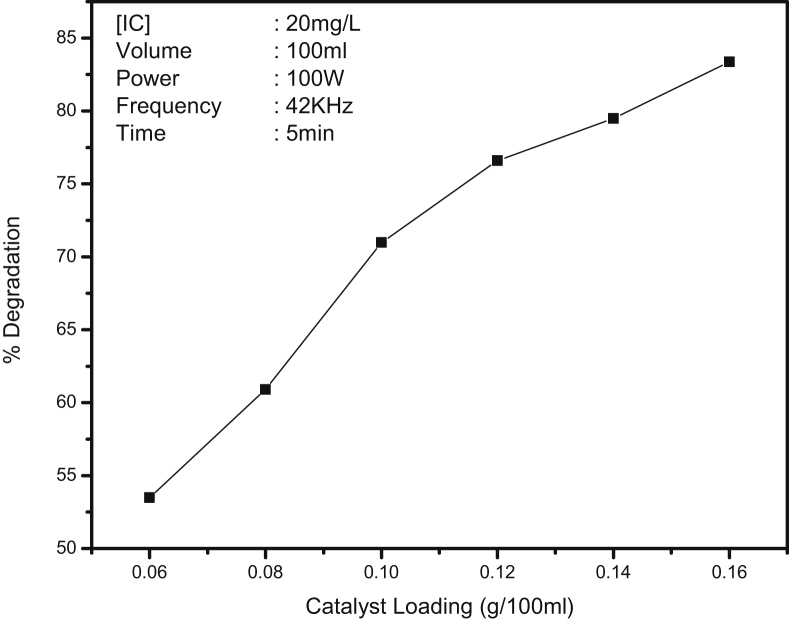
Effect of catalyst loading on the sonocatalytic degradation of IC.

The effect of dosage of MnO_2_ on the sonocatalytic degradation of IC under otherwise identical conditions is shown in Fig. 3. The degradation increases with increase in catalyst loading. Unlike in the case of many other AOPs, the degradation does not have a clear optimum with respect to catalyst loading and continues to increase with increase in the dosage. However, the rate of increase slows down above 0.14 g of MnO_2_ when the degradation has reached ∼80%. At this loading, which is taken as convenient optimum for further studies, the rate constant is about 1.6 × 10^−1^ min^−1^. The degradation under identical condition is practically negligible without MnO_2_.

The increasing degradation efficiency with increase in loading is probably due to increased number of adsorption sites and more effective absorption of US which lead to enhanced cavitation and consequent effects. This leads to the formation of higher number of reactive hydroxyl radicals and their interactions with the dye. The aggregation of catalyst particles which causes decrease in the number of available active surface sites and consequently decrease in the activity, observed in the case of other AOPs such as photocatalysis, sets in only at a much higher dosage in the case of sonocatalysis. This is because the US is deaggregating the agglomerated particles and thus increasing the surface area of the catalyst constantly. Hence the increase in sonocatalytic activity with catalyst dosage can be sustained even at higher loadings of MnO_2_ compared to the ‘dosage effect’ in other AOPs. Presence of particles is also known to provide additional sites for the cavitation phenomena resulting in increased number of cavitation events and hence increased reaction [17, 22, 23]. With the reactants remaining adsorbed on MnO_2_, the cavitation effect will be more efficiently transferred on to the molecules resulting in enhanced degradation. The adsorption of IC on MnO_2_ under the optimized degradation conditions is experimentally determined and is ∼30 %. Hence adsorption of the substrate is an important factor that contributes to the higher sonocatalytic efficiency of MnO_2_. The activated molecules can degrade more efficiently and then get desorbed from the surface under US thereby making the surface continuously available for fresh molecules to get adsorbed. The sonocatalytic degradation is the net effect of simultaneous adsorption, desorption and actual degradation of the pollutant. This adsorption-desorption process is important though the degradation of the pollutant takes place mainly in the bulk. This is proven from the observation that even coal ash with relatively low catalytic activity enhances the sonocatalytic degradation of phenol [48]. Similar results are reported by Tuziuti et al. also [49]. Suspended particles can also help the break-up of the micro bubbles created by US into still smaller ones thereby increasing the number of regions of high temperature and pressure [46, 47]. This leads to increase in the number of ^·^OH radicals which can interact with the organic pollutant molecules present in water and oxidise them into various intermediates resulting in eventual mineralization. The surface of the suspended particles can also act as nucleation sites for cavitation bubbles [50]. Enhancement in the sono induced destruction of chemical and bacterial pollutants in the presence of suspended particles of Al_2_O_3_, TiO_2_ and ZnO have been reported [4, 6, 51].

Another factor contributing to the higher optimum dosage of catalyst may be the US- induced increase of the mass transfer between the liquid phase and the catalyst surface [52], making the surface consistently and more readily available for fresh reactant molecules to get adsorbed and interact. The continued increase in degradation with increase in catalyst dosage and the absence of any optimum can also be explained based on the effects of microstreaming and increased mass transport induced by the interaction of US with solid matter. Microstreaming causes a jet of fluid directed onto the particle [18, 53] and this will clear the blockage of active adsorption sites of the catalyst partially or even fully. Consequently, the catalyst surface is regenerated insitu which helps recycling of the catalyst. Under normal reaction conditions, MnO_2_ can be recycled at least three times without loss of activity. However, the results are different in reaction systems deoxygenated by N_2_ bubbling as demonstrated in Section 3.7.

The dynamic factors discussed above make the particle size variation of the catalyst within short to medium range less important in sonocatalysis [8]. In any case, the particles cannot be fully and effectively suspended beyond a particular loading in a particular reactor which leads to suboptimal penetration of US and reduced adsorption of the substrate on the surface. Excess MnO_2_ may also be deactivating at least a part of the originally activated catalyst by collision with ground state particles according to the equation [54].

(4) MO* + MO ⟶ MO^#^ + MO

MO represents the suspended MnO_2_ particle, MO* and MO^#^ are the active and deactivated forms respectively.

At higher loading of the catalyst, the effective working volume of the suspension will be low [47] and this can affect the penetration of US through the dense medium. Consequently, the enhancement due to the deagglomeration of particles may be balanced or even negatively impacted. Hence any further enhancement in degradation beyond a particular catalyst loading will be slow unless the US power is increased significantly. In the current study also, enhancing the US power from 50 to 100 W at the selected optimum catalyst loading of 1.4 g/L, under otherwise identical conditions, increased the degradation of IC from 30.7 to 38.5% in one minute. Davydov et al. [55] also reported similar findings. The slowdown in the rate of degradation at higher catalyst dosages can also be due to the increased scattering of the incident sound waves thereby decreasing the available energy for cavity formation [56].

The optimum catalyst dosage is also dependent on the reactor geometry and operating conditions; in particular the power dissipation into the reactor in the case of sonocatalysis. Hence, the optimization has to be made individually for each reactor configuration.

### 3.2 Effect of initial concentration of IC

**Fig. 4:**
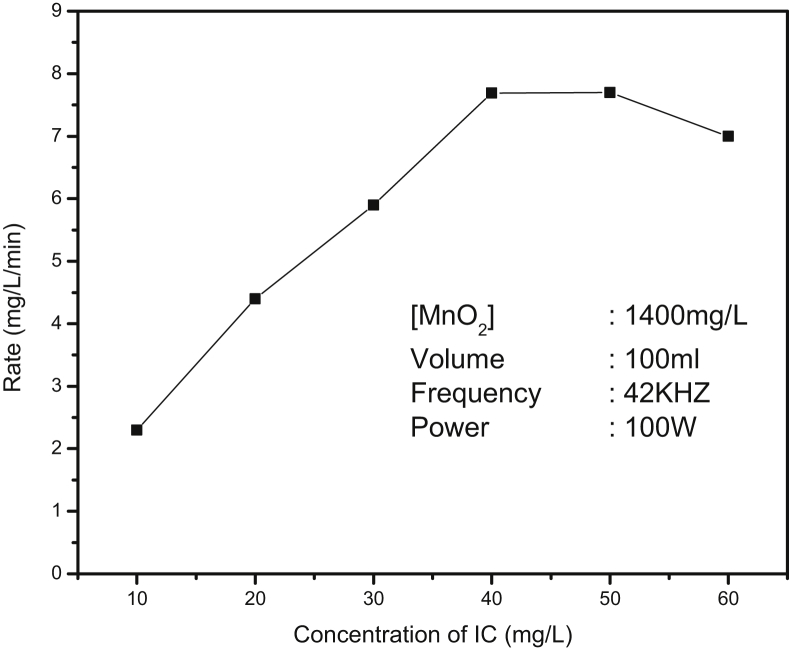
Rate of degradation of IC at various concentrations.

The effect of initial concentration of IC in the range of 10–60 mg/L on its sonocatalytic degradation in presence of MnO_2_ is investigated. The % degradation decreases with increase in concentration of IC. However, it will be more appropriate to compare the rate of degradation to evaluate the concentration effect. The rate vs concentration plot is shown in Fig. 4. The degradation rate increases linearly with increase in concentration in the range 10–40 mg/L. At higher initial concentration (>40 mg/L) the rate remains stable or even slows down slightly thereby demonstrating that the factors favoring degradation of IC under sonolysis are less effective at higher concentration. This also confirms the variable kinetics reported in the case most AOP initiated degradation of water pollutants [30, 57]. The optimum rate of degradation under the current experimental condition is 7.5 mg/L/min.

Sonocatalytic degradation reactions taking place at the solid–liquid interface can be represented by the modified Langmuir-Hinshelwood (L–H) model [58, 59]. Accordingly, the rate of degradation of IC in the present case, i.e., r_0_ (mg L^−1^ min^−1^) may be presented as:

(5) r_0_ = −dC/dt = k_r_KC_0_/ (1 + KC_0_)

where *k*_*r*_ is the reaction rate constant at maximum surface coverage, K is the equilibrium adsorption coefficient and C_0_ (mg L^−1^) is the initial concentration. The assumption is that the reaction intermediates or byproducts are not interfering with the interaction between substrate and the surface. Eq. (5) may be rewritten as

(6) 1/*r*_*0*_ = 1/k_r_ + 1/k_r_K (1/C_0_)

**Fig. 5:**
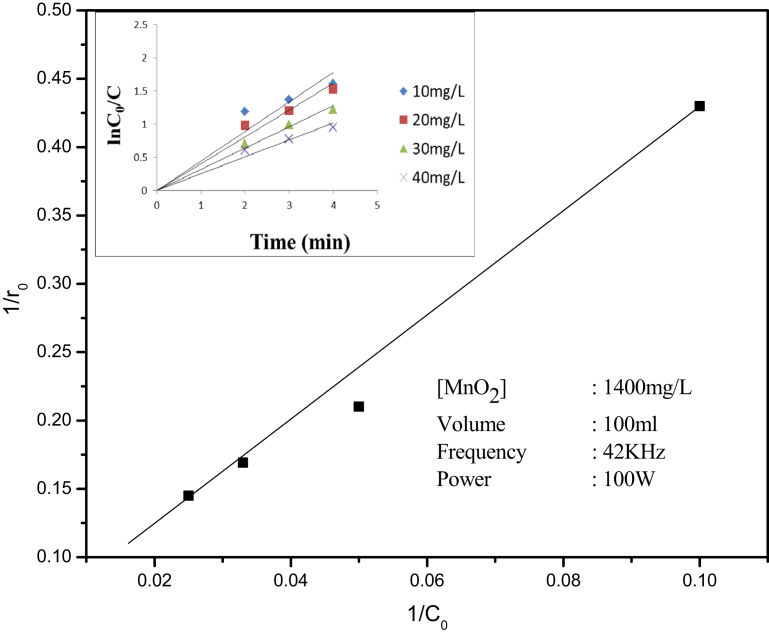
Reciprocal plot of initial rate of degradation of IC versus its initial concentration. [Inset]: Logarithmic plot of pseudo first order for the degradation of IC.

The inverse plot (1/*r*_*0*_ vs 1/C_0_) yields straight line in the concentration range 10–40 mg/L which indicates first order kinetics and L–H mechanism (Fig. 5).

Eq. (5) on integration and rearrangement yields;

(7) ln (C_0_/C) + K (C_0_-C) = k_r_Kt

When C_0_ is very small, Eq. (7) becomes

(8) ln (C_0_/C) = k_r_Kt = *k′t*

The plot of ln(C_0_/C) versus irradiation time (t) yields straight lines (Fig. 5 inset) for the concentration range of 10–40 mg/L thereby reconfirming pseudo first order kinetics. The slope of each line gives the apparent rate constant of degradation *k′* for respective concentration of the substrate. The rate constants computed are given in Table 1.

**Table 1:** Pseudo first order rate constants for the sonocatalytic degradation of IC over MnO_2_.

Sl. No.	MnO_2_ (g/L)	IC (mg/L)	k′ × 10^−1^(min^−1^)
1	1.4	10	6.4
2	1.4	20	4.3
3	1.4	30	3.0
4	1.4	40	2.4

The rate constant decreases with increase in concentration of IC. The relative decrease is more in the lower concentration range (∼33% in the range 10–20 mg/L, ∼20% in the range 30–40 mg/L) which may be rationalized as follows: For specific reaction conditions, including fixed amount of catalyst, the number of surface-generated active species available for interaction also will be finite. Hence at higher concentrations, the number of substrate molecules is relatively excessive. Hence the relative % fraction of the substrate which can successfully interact with the ROS will be less resulting in decrease in the apparent rate constant. However the absolute values of rate constants are relevant only under specific experimental conditions and cannot be generalized.

Decrease in the rate of degradation with increasing concentrations of the substrate beyond the optimum and consequent decrease in the order of the reaction has been reported in the case of many AOPs including sonocatalysis and sonophotocatalysis [56, 57]. However, Davydov et al. [55] reported that the TiO_2_ mediated sonophotocatalytic degradation of salicylic acid follows zero order kinetics. In this case, the study was conducted at relatively higher concentration of the substrate and hence in the lower conversion regime only. In the current instance too, the shift in kinetics from first order to zero order takes place only at higher concentration of the substrate and hence lower % conversion ranges.

The first order kinetics at lower concentrations of IC and higher % conversion ranges can be explained from the effect of sonication on the catalytic role of MnO_2_. With increase in the concentration of IC, more molecules get adsorbed on the catalyst site, get activated and interact with correspondingly more reactive oxygen species (ROS) including ^·^OH radicals (as explained under Section 3.11). This increase will continue until all the surface sites are occupied. Thereafter, the rate of degradation is stabilized or even decreases. Similar stabilization/decrease takes place in the bulk also where the relative (to the substrate) concentration of OH radicals is smaller [60]. Higher concentration of the dye can utilize the limited ^·^OH radicals in the bulk more effectively leading to increased degradation. This will continue until the IC concentration is sufficiently high to interact with optimum number of ^·^OH radicals. Thereafter the rate of degradation is independent of the increase in IC concentration and the reaction is of zero order. Also, at higher substrate concentration, it will absorb at least part of the US radiation thereby decreasing the energy available for the catalyst activation and the formation of ROS.

The above factors together can contribute to stabilization and decrease in the rate of degradation. However, due to the US induced deaggregation and consequent reduction in size and change of shape of the catalyst particles, there will be insitu formation of newer surface sites. Further, the reaction can also take place at the cavitation bubble interface where the OH concentration can reach a higher limit [61]. Hence, the initial degradation rate of IC will be dependent on its concentration on the surface, in the bulk and at the interface of the cavitation bubble as well as the concentration of ^·^OH radicals. Consequently, the catalyst dosage and substrate concentration for optimum degradation will be higher compared to other AOPs. This optimum may also depend on a number of other parameters such as frequency and power of the US radiation, mass, type and physicochemical characteristics of the sonocatalyst, type, size and geometry of the reactor etc. Consequently any optimization will apply only to the particular reaction conditions and it cannot be generalized.

When the concentration of the dye is small, many of the ^·^OH radicals will get deactivated by interaction among themselves resulting in the formation of H_2_O_2_ (reaction 36). At higher IC concentration, the probability of interaction of the dye with ^·^OH is more and the rate of degradation increases until the optimum. Consequently, the concentration of H_2_O_2_ is often less at higher concentration of the substrate [5] though other factors also contribute to this as discussed below.

### 3.3 Formation and fate of H_2_O_2_

H_2_O_2_ is an essential byproduct of many AOPs, especially of those based on sono- and photo-catalysis. Hence, the effect of concentration of IC on the net amount of H_2_O_2_ present in the system is verified experimentally. The quantity of H_2_O_2_ present in the system immediately on decolorization is measured at different initial concentrations of IC and the results are shown in Table 2. (The concentration of H_2_O_2_ was not measured during the decolorization, when the solution was still colored, due to constraints of the analytical procedure followed).

**Table 2:** Effect of concentration of IC on the net amount of insitu formed H_2_O_2_ in the system measured immediately after decolorization.

MnO_2_: 1.4 g/L		
[IC] mg/L	Time for decolorization (min.)	[H_2_O_2_] mg/L
10	15 min	12.0
20	20	6.7
30	35	7.8
40	45	5.4
50	60	7.4
60	80	6.4
70	120	7.6

Obviously, the concentration of H_2_O_2_ does not consistently increase with increase in concentration of the dye or increasing rate of degradation. At the optimum concentration of the dye (in this case >40 mg/L) its degradation rate is stabilized. However the concentration of concurrently formed H_2_O_2_ is not steady thereafter as expected and continues to increase and then fluctuates with the concentration (of IC).

**Fig. 6:**
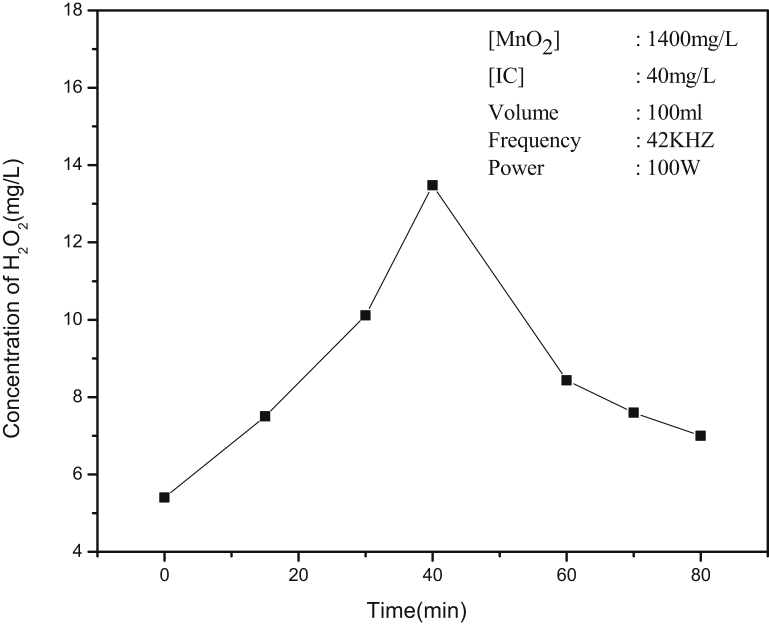
Variation in the concentration of in situ formed H_2_O_2_ after decolorization of IC with time irradiation. Time starts (0) immediately on decolorization.

The concentration of H_2_O_2_ at different times of irradiation in the combined presence of MnO_2_ and IC (after the decolorization of the dye) is measured and the data is given in Fig. 6. Even after the decolorization of IC, the degradation of various insitu formed intermediates (before they eventually get mineralized) will continue and correspondingly, the concentration of H_2_O_2_ in the system must also be increasing and then stabilizing. However, in this case, the increasing trend is observed only upto 45 minutes and thereafter the concentration of H_2_O_2_ decreases with time indicating concurrent competitive formation and decomposition reactions as reported earlier [5]. The inconsistency and poor reproducibility in the concentration of H_2_O_2_ at any point of time during the US irradiation (though the fluctuating trend remains consistent) can be attributed to the phenomenon of ‘oscillation’ (in the concentration of H_2_O_2_) [5, 57].

When the relative concentration of the dye is less, the reactive ^·^OH radicals will interact among themselves more frequently rather than with the dye resulting in higher concentration of H_2_O_2_ and lower degradation of IC. The ^·^OH radicals will also interact with the H_2_O_2_ leading to the decomposition of the latter (reaction 27). The relative rates of the multiple competitive interactions in the system will depend on various reaction and system parameters and the dominating reaction at any point in time will determine the net concentration of H_2_O_2_ at that moment [5]. Another factor which leads to the inconsistency in the concentration of H_2_O_2_ is its thermal decomposition (which may occur in the vicinity of cavitation bubble where extreme local conditions exist) to water and oxygen rather than to reactive radical species. Due to all these factors, there is no direct correlation between the degradation of IC and the net concentration of H_2_O_2_ in the system.

### 3.4 Effect of pH on the degradation of IC

**Fig. 7:**
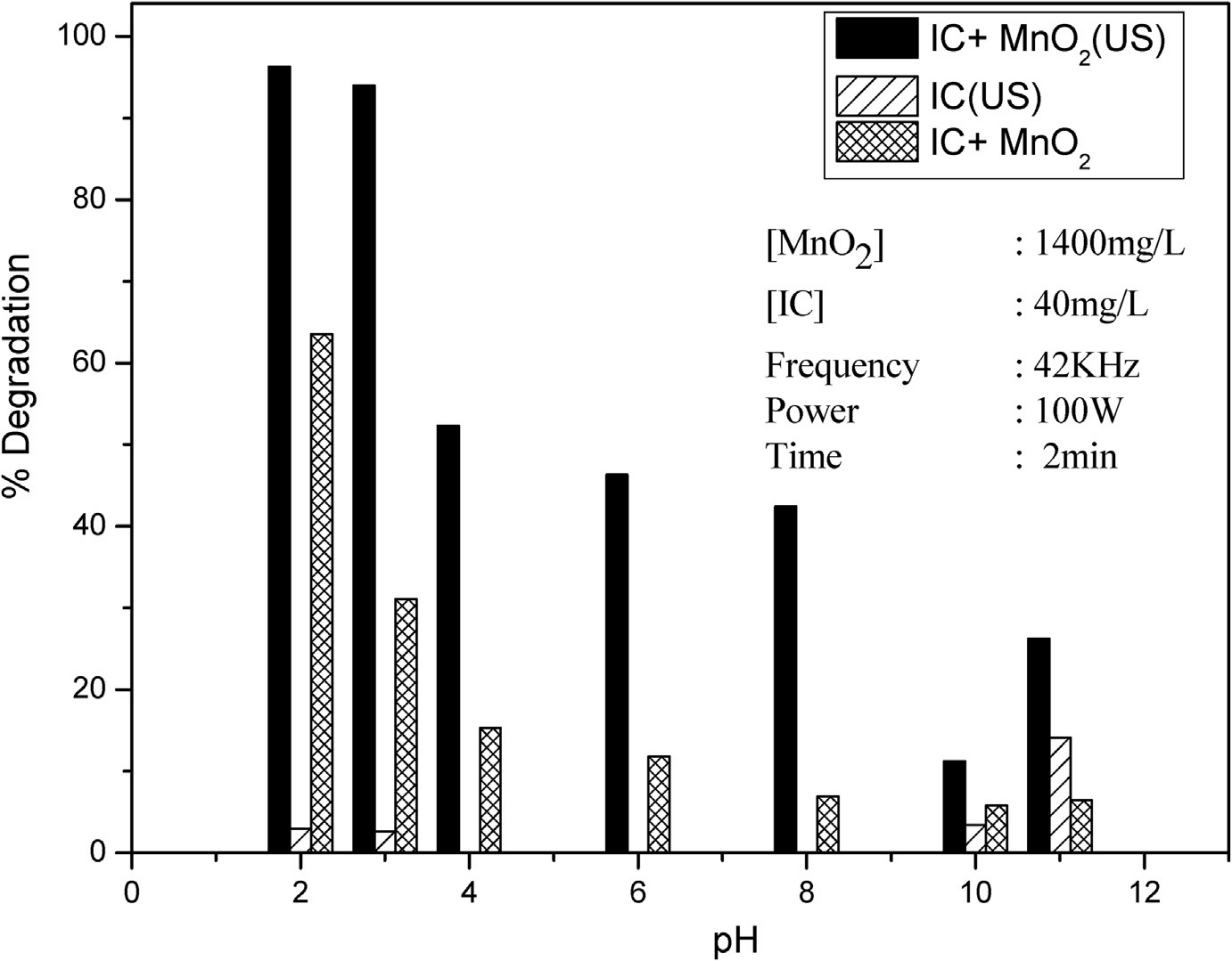
Effect of pH on the sonocatalytic degradation of IC in the presence and absence of MnO_2_.

The AOP-induced degradation of organic pollutants in water is often dependent on the pH of the solution. The effect of pH on sonocatalytic degradation of IC in presence of MnO_2_ is investigated in the range 3–11. The pH of the suspension was adjusted initially and was not modified externally during the irradiation. The results are presented in Fig. 7. The degradation is the highest at pH 2–3, decreases sharply from pH 3 to 4 and remains fairly steady or decreases slightly thereafter from pH 4 to 8. This is followed by another sharp decrease at pH 10 and slight increase at pH 11. Experiments with the dye solution under identical conditions, but without MnO_2_ show practically ‘no degradation’ with or without US in the pH range of 2–10. However, in this case also there is sharp increase in degradation at pH 11. The pH effect in presence of MnO_2_ without US irradiation is also shown in the figure. The degradation is more in presence of MnO_2_ and US irradiation at all pH. However, the trend of ‘pH effect’ remains qualitatively the same in the presence or absence of irradiation. This indicates that the effect of pH on the physicochemical and surface characteristics of MnO_2_ is primarily responsible for the ‘pH effect’ on the sonocatalytic degradation.

The pH of the reaction medium influences the characteristics of the inorganic semiconductor oxide particles such as the surface charge, size of the aggregation and the band edge position. Hence pH can affect the adsorption – desorption properties of the catalyst-substrate combination. In addition to affecting the surface properties of the catalyst, pH also influences characteristics of the organic molecules and the reactive ^·^OH radical formation [62]. Alkaline range is expected to favor the formation of more ^·^OH radicals from the large quantity of OH ions present which could have enhanced the degradation significantly. However this is not fully reflected in the actual degradation rate possibly due to the poor adsorption of the dye as explained below.

The effect of pH on the degradation of organic pollutants in presence of semiconductor oxides is often explained based on the point of zero charge (PZC) of the solid [8, 9]. The PZC of MnO_2_ is ∼4.7 [30]. Below this pH value the surface of the catalyst is positively charged and hence its oxidizing ability will be relatively higher. IC is a dianionic dye in aqueous solution and this configuration is maintained in the pH range 3–11 [28, 29]. The electrostatic interaction between the positively charged MnO_2_ surface and the dianions of IC will be strong below the PZC leading to strong interaction/adsorption and subsequent reaction. MnO_2_ induced heterogeneous degradation is known to involve the formation of a precursor complex between the surface bound Mn (IV) and the organic contaminant by the transfer of electrons from the latter to the former [63]. This results in the oxidative degradation of the organic compound. Concurrently the Mn(II) is reductively dissolved in the bulk solution. Thus MnO_2_ plays the dual role of a catalyst as well as the oxidant. The Mn(II) is oxidized to Mn(IV) oxide again by the O_2_ dissolved in the solution. Here MnO_2_ functions as a catalyst and dissolved O_2_ is the oxidant. The re-adsorption of free Mn(II) ions in solution back onto MnO_2_ surface is facilitated at pH > PZC when the surface is negatively charged. This in turn results in decreased generation of reactive species and consequently lower degradation at higher pH. However, the steep decrease in degradation in presence of MnO_2_ at pH ∼ 4, below the PZC of 4.7, reveals that it is not the surface initiated process alone that is important in the sonocatalytic degradation of IC. The influence of pH on other parameters, especially the bulk processes, is equally important.

The dramatic increase in the degradation of IC at low pH ≤ 3 has been reported in the case of the photocatalytic oxidation of the dye in presence of Mn supported TiO_2_ [28]. This happens even without irradiation and can hence be at least partly due to pH induced interactions/transformations of the catalyst. However, US plays its own accelerating role, as in the case of many US initiated processes, once the reaction is initiated. Since IC keeps its anionic configuration upto around pH 11 it cannot get adsorbed or even come closer to the catalyst above its PZC of 4.7. This may be the main reason for the relatively lower degradation of the dye at higher pH. However reasonable degradation at all pH ranges shows that other parameters are also relevant. The lack of direct correlation between the PZC of MnO_2_ and the ‘pH effect’ can also be attributed to the fact that the PZC itself is not rigid and depends on a number of factors including the size and nature of dispersion of the particles and the type of catalyst itself. It has also been reported that the zeta potential curve may remain positive over a wider range under sonication [64, 65]. The alleviation of pH dependence of MnO_2_ mediated degradation of IC under microwave irradiation has also been reported [28, 29]. In any case, the pH effect is very complex and depends on the interplay of many factors in the system which cannot be explained based on individual reaction parameters in isolation.

### 3.5 Volume effect

**Fig. 8:**
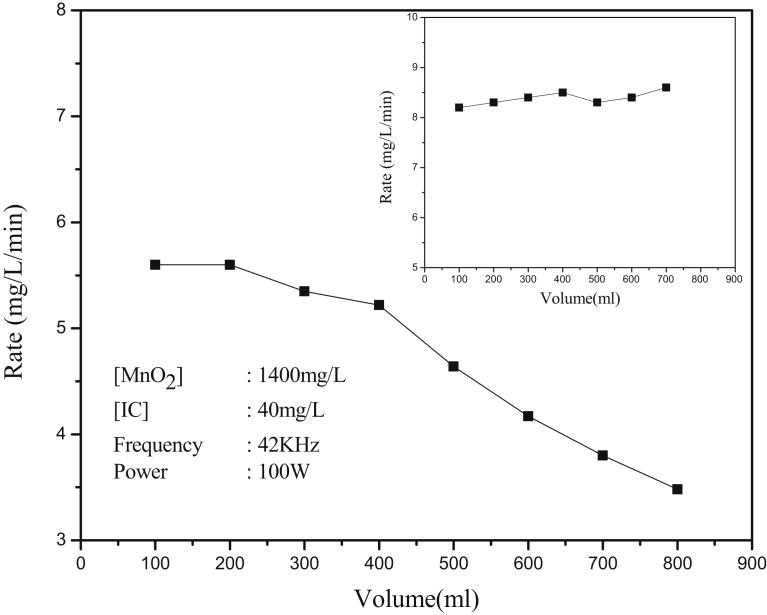
Effect of volume (at constant MnO_2_ dosage) on the degradation of IC. [Inset]: Effect of volume, with correspondingly varying MnO_2_ dosage, on the degradation of IC.

Chen and Smirniotis [47] and Anju et al. [66] reported that in the case of semiconductor oxide catalysts such as TiO_2_ and ZnO, reducing the volume of the reaction suspension, without changing other parameters, results in dramatic increase in the synergistic effect of sonophotocatalysis over individual sono and photocatalysis. In this context, the effect of reaction volume on the MnO_2_ catalyzed sonocatalytic degradation of IC is examined. The results are presented in Fig. 8. It is seen that under otherwise identical conditions, the degradation rate decreased slowly with increase in the reaction volume. This is partially due to the increase in the thickness of the irradiated region which in turn increases the attenuation of US intensity through the reaction medium. The negative effect of increasing volume can also be due to the relatively lower availability of catalyst particles (which is kept constant) per molecule of the dye for effective interaction. The possibility of compensating for this by keeping the catalyst-substrate ratio in the suspension constant by suitably varying the catalyst dosage is examined. The results are presented in the inset of Fig. 7. In this case the rate remains practically unaffected by changing volume indicating that the higher dosage of MnO_2_ generates correspondingly more ^·^OH radicals to interact with the increasing number of IC molecules at higher volume. The decreasing rate at higher volume can also be due to the absorption of ultrasonic energy by the surrounding apparatus, i.e. the reactor wall, cooling water and solvent/medium (in this case water). The results clearly show that reaction conditions, especially the catalyst-substrate ratio, reactor geometry and size are important in deciding the volume effect.

In the case of sonophotocatalysis, it has been reported that the decreasing reaction rate at higher volume can be reversed by applying correspondingly more power which results in increase in the number of active cavitation bubbles and consequent generation of more ^·^OH radicals [47, 66]. However, application of excessive power can disrupt the bubble dynamics resulting in abnormal bubble growth and poor cavitation. Hence proper correlation between frequency and power of US coupled with volume of the reaction medium and catalyst dosage is important in balancing the bubble growth and achieving optimum efficiency.

### 3.6 Effect of frequency

In addition to the role of power discussed above, another US parameter that can influence the sonication effect is the frequency. Preliminary study of the effect of frequency of US on the degradation of IC is verified at 42 and 53 kHz, keeping the power constant at 100 W. The degradation decreases by 15% with increase in frequency from 42 to 53 kHz. Frequency of US influences the pressure cycle and causes high turbulence in the liquid [67, 68, 69]. This effect known as acoustic streaming dominates acoustic cavitation at high frequency and amplitude. Consequently the bubbles die prematurely resulting in poor cavitation and material growth. Increasing the frequency also reduces diffusion time which slows down the growth of cavitating bubble resulting in smaller bubbles and uniform distribution. Hence the rate and type of radical formation is dependent on frequency though there is no linear relation between the two. However the effect of frequency on the cavitation and subsequent processes cannot be generalized as it depends on the characteristics of the reaction system. Pending further investigations on frequency and power effect, in the current instance, all investigations are made at 42 kHz.

### 3.7 Effect of dissolved oxygen

Presence of dissolved gases increases the cavitation threshold and hence adversely affects the sonocatalytic degradation efficiency. However, small amount of dissolved gases is necessary to effect efficient cavitation. The presence of dissolved O_2_ during ultrasonic irradiation is known to play significant role in the formation of reactive ^·^OH radicals which are primarily responsible for the degradation of the pollutant. This role of O_2_ is verified by measuring the sonocatalytic degradation of IC in MnO_2_ systems deaerated with N_2_. The degradation of IC is practically unaffected by deaeration thereby suggesting that the main source of O_2_ for the reaction is not the dissolved form. MnO_2_ has strong affinity towards O_2_. It is also a rich reservoir of O_2_. Hence significant amount of O_2_ will be present in the MnO_2_/IC suspension, even after long deaeration. Further, it is known that in the case of MnO_2_ mediated photocatalytic and microwave catalytic reactions, it functions not only as a catalyst but also as a powerful oxidant [29, 30]. The required O_2_ is provided primarily by the surface (adsorbed) and lattice of MnO_2_ which cannot be easily removed by flushing with N_2_. The participation of lattice oxygen which makes MnO_2_ deficient in oxygen both atomically and by weight, is demonstrated by comparison of the Energy dispersive X-ray (EDX) spectrum of the fresh and used catalysts.

If the effect of deoxygenation of the suspension by N_2_ is compensated by the lattice and strongly bound O_2_ in MnO_2_ the effect will be dependent on the dosage of the catalyst, i.e. the less the amount of MnO_2_, the more the effect of deaeration by N_2_. This is verified by measuring the deaeration effect on the sonocatalytic degradation of IC at different dosages of MnO_2_. The results are given in Table 3.

**Table 3:** Effect of MnO_2_ dosage on the degradation of IC in systems deaerated with N_2_. N_2_ bubbling time: 60 min. [IC]: 60 mg/L.

Sl. No.	MnO_2_ dosage mg/L	Reaction time (min.)	% Degradation		Effect of N_2_ bubbling on the degradation
			Standard	After N_2_ bubbling	
1	1400	10^a^	74.5	76.2	+2.2% ∼ No change
2	1000	15^a^	62.8	64.3	+2.4% ∼ No change
2	200 mg/L	30	12.4	11.3	−8.8%, Inhibition
3	100 mg/L	30	9.6	6.8	−29.2% Inhibition
4	50 mg/L	30	5.6	2.6	−53.6% Inhibition

a Lower time is taken because the degradation was complete by 30 minutes.

At lower MnO_2_ dosage the deaeration by N_2_ decreases the degradation probably because the O_2_ available from the MnO_2_ is not adequate to compensate for the removed O_2_. With increase in the quantity of MnO_2_, more O_2_ is available to compensate for the loss by deaeration and hence the effect is also less.

Under US irradiation, the O_2_ present in the solution/suspension would be split into reactive ^·^O radicals which interact with H_2_O molecules (reaction 9) and form reactive ^·^OH. The ‘hot spot effect’ of US irradiation in water enhances the formation of ^·^OH due to the pyrolysis of water molecules as in reaction (3). This is followed by the generation of more free radicals as in reaction 9.

(9) O + H_2_O → 2 ^·^OH

(10) H^·^ + O_2_ → O + ^·^OH

Additionally, the US induced ‘sonoluminescence’ [22, 23] generates light of wide wavelength range which can excite the semiconductor oxide and make it a potential photocatalyst capable of generating electron-hole pairs. This also leads to the production of reactive ^·^OH radicals as below:

(11) MnO_2_ + hν → h^+^ + e^−^

(12) e^−^ + O_2_ → O_2_^−·^

(13) h^+^ + H_2_O → ^·^OH + H^+^

Thus the unproductive recombination of the electrons and holes is also prevented in the presence of O_2_. The presence of dissolved gases will also create deformities in the medium which eases the generation of cavitation events [70]. Hence it is reasonable to assume that the presence of O_2_ is essential to initiate and enhance the degradation under US irradiation. During the process of sonication, when the cavitation induced bubbles break, temperature rises and some of the dissolved gases will be released. Hence an optimum amount of O_2_ may be required for efficient generation of reactive free radicals and consequent sonocatalytic degradation. Irrespective of whether the US induced molecular activation is thermal and/or electrical, the molecules are brought into excited state and dissociate in the interior of the bubble cavities filled with gas (N_2_/O_2_/air) and/or vapour.

Various steps leading to the formation of reactive ^·^OH radicals in presence of O_2_ under US irradiation were explained earlier. Further, even in deaerated system, the residual O_2_ present in the gas bubble scavenges the H^·^ as:

(14) H^·^ + O_2_ → HO_2_^·^

(15) 2HO_2_^·^ → H_2_O_2_ + O_2_

The detection of moderate amounts of H_2_O_2_ even in the deaerated system confirms the presence of this reaction.

In the presence of MnO_2_, more interactions with H_2_O_2_ can occur;

(16) MnO_2_ + H_2_O_2_ + 2H^+^ → Mn^2+^ + 2H_2_O + O_2_

Thus O_2_ is regenerated insitu in the system in presence of MnO_2_, even though part of it is consumed for the generation of ROS including H_2_O_2_. Hence the effect of deaeration by N_2_ is not significant at higher dosage of MnO_2_.

Amorphous MnO_2_ is known to release bulk oxygen more easily to the surface which makes it a better catalyst in terms of facile activation and regeneration [30, 32]. MnO_2_ with multiple oxidation states together with its electron donor-acceptor properties is an excellent oxidation – reduction catalyst. Sonolysis of MnO_2_ and resultant photoluminescence and consequent photolysis and sonophotolysis increase the number of oxygen species on the surface either by oxygen migration to the surface or by Mn migration to the bulk or both. Surface oxygen is consumed faster upon irradiation and oxygen from the bulk moves to the surface. In the case of MnO_2_, the loss of oxygen takes place at temperatures as low as 50 °C. The sono/photo-initiated oxygen release from MnO_2_ may be due to movement of O^2−^ (bulk) to the surface and subsequent weakening of MnO_2_ bonds [56]. In the re-oxidation of oxides, atmospheric oxygen and/or dissolved O_2_ is taken up by the surface of the partially reduced MnO_2_ as follows:

(17) O_2_ (g) + e^−^ → (O_2_^−^) g-gas

(18) (O_2_^−^) + e^−^ → 2(O^−^) → O^2−^ (s) s-surface

Most of the oxygen radicals are present in the bulk of the catalyst while some of them may remain on the surface for a short period. These highly active oxygen radicals can regenerate reduced manganese species. Under US irradiation, the bonds in MnO_2_ are weakened and O^2−^ is released to the surface. Even though the lifetime of excited state oxygen species is short, they possess adequately high energy and electronegativity to facilitate the reduction or hydrogen abstraction from the substrate. Possible reaction pathways are:

(19) $${\text{O}}^{2-}\text{(s)}\overset{\text{h}\nu }{\underset{{–e}^{-}}{\to }}{\text{(O}}^{-}\text{)}\overset{\text{h}\nu }{\underset{{–e}^{-}}{\to }}{\text{O}}_{2}\text{(s)}$$

(20) O_2_ (s) + RH (i.e., IC) → HO_2_^·^ (s) + R^·^

(21) O_2_ (s) + R^·^ → Intermediates → → → Mineralization (CO_2_ + H_2_O)

The abstracted H^·^ interacts with the hole and gets oxidized to form acid sites on the surface of MnO_2_.

(22) H^·^ + h^+^ → H^+^

The regeneration of MnO_2_ may be represented as:

(23) $$-Mn^{\left(3+\;or\;2+\right)}\overset{\text{hν}}{\underset{{\text{e}}^{-}}{\to }}{-\text{Mn}}^{\text{(}4+\;\text{or}\;3+\text{)}}$$

(24) n(−Mn^(4+ or 3+)^−) + O^2−^ (from reaction 18) → MnO_2_ (or Mn_3_O_4_)

Unless the oxygen is replenished periodically, the activity of the catalyst will be lost faster. This is verified by recycling the catalyst immediately after use in the N_2_ flushed system. The used catalyst is separated by simple filtration followed by quick drying at 120 °C for 1 hr. The degradation of IC (in 30 minutes) decreased steeply from ∼75% in presence of fresh catalyst to ∼30% in first recycling, ∼11% in second recycling and ∼8% in third recycling. This confirms drastic change in the surface characteristics, loss of adsorption sites and loss of oxygen from the lattice, bulk and/or the surface of MnO_2_ during the reaction which are not fully compensated by contact with atmospheric oxygen. The migration of bulk oxygen to the less energetic surface sites and its consumption by participation in catalytic reactions has been proven experimentally and from ESR studies [71].

The effect of replacement of air at least partially by N_2_ or inert gases can be even more complex and unpredictable. When the dissolved air is replaced by degassing with N_2_, the system will still contain moderate amounts of both N_2_ and O_2_. As explained earlier, O_2_ is available from MnO_2_ even in deaerated solution. The high temperature and pressure conditions of sonolysis will induce interaction between both these gases resulting in the formation of nitrogen oxides, nitrate, nitrites and even the unstable peroxy species peroxynitrous acid [72, 73]. These species will lead to a number of even more complex reactions under sonication, that too in presence of the highly active catalyst MnO_2_. Substitution of air by N_2_ can also modify the maximum temperature reached by the collapsing bubbles and the rate of heat dispersion. The facile ^·^OH generation by sonolysis in presence of O_2_ will also be slowed down.

The presence of gases in dissolved form or as individual bubbles is known to provide additional nuclei for the generation of cavitation leading to enhanced number of cavitational events. This will enhance the pressure/temperature pulse generated in the system and the formation of reactive free radicals. However, too much of aeration (or of any gas) will result in decoupling effect leading to decreased energy input into the system [7]. Generation of large number of gaseous cavities is often not advantageous because the energy content of these cavities will be substantially lower compared to that of the vaporous cavities. The effect of the presence of gases can be further complicated because the physicochemical properties of the gas such as solubility, thermal conductivity, polytropic index etc. can affect the cavitation intensity. Hence in order to achieve maximum sono degradation of the pollutant, the aeration/gassing of the reaction systems has to be optimised with and/or without catalyst, especially so in the presence of a strong O_2_ reservoir such as MnO_2_.

### 3.8 Effect of oxidants

Oxidants such as H_2_O_2_, persulphate (PS), Chromate, permanganate, periodate etc. are known to enhance the AOP degradation of many organic pollutants in water. In this context, the effect of two typical oxidants, i.e. H_2_O_2_ and PS on the sonocatalytic degradation of IC in presence of MnO_2_ is investigated and the results are as follows:

#### 3.8.1 Effect of added H_2_O_2_

**Fig. 9:**
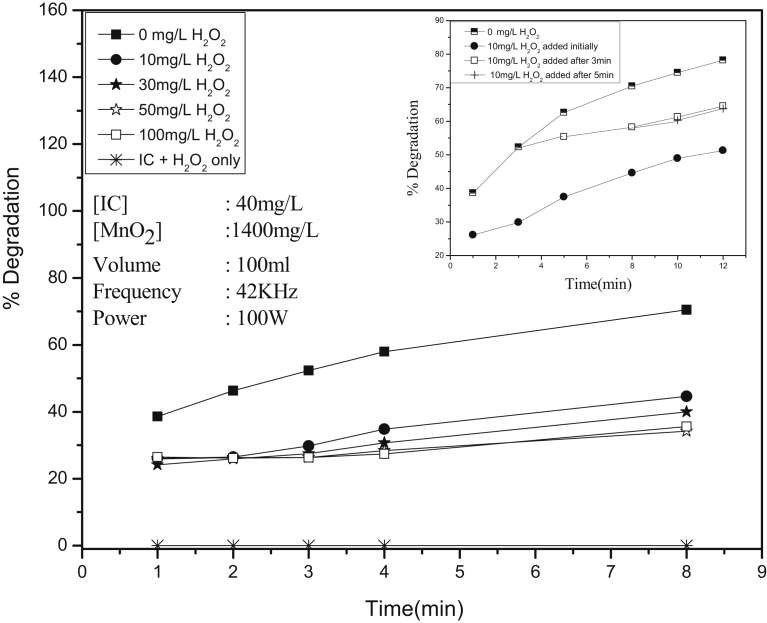
Effect of H_2_O_2_ on the Sonocatalytic degradation of IC over MnO_2_. [Inset]: Effect of initial and in between addition of H_2_O_2_ on the degradation.

Effect of added H_2_O_2_ on the sonocatalytic degradation of IC is tested in presence of MnO_2_. The results are shown in Fig. 9. Contrary to expected enhancement, the degradation is inhibited by H_2_O_2_ even at low concentration of 10 mg/L. Further increase in H_2_O_2_ increases the inhibition only slowly and even stabilizes with further increase. This is consistent with the results on the contradicting effect of H_2_O_2_ reported in many earlier studies according to which H_2_O_2_ can be an enhancer or inhibitor depending on the reaction conditions. The inhibition effect is further verified by in-between addition of H_2_O_2_ to the reaction system at different time intervals. In this case, the inhibition of the degradation begins from the moment of addition of H_2_O_2_ (Fig. 9 inset).

Under US/MnO_2_ conditions, H_2_O_2_, which is an oxidant, undergoes decomposition. All the reactive oxygen species, including the powerful ^·^OH radicals, thus generated do not interact exclusively with the IC molecules, because, had it been so, the degradation of IC should have been enhanced. It is possible that under these conditions the following reactions may be taking place:

(25) $$2H_{2}{\text{O}}_{2}\;\overset{\text{US}}{\to }\;2{\text{H}}_{2}\text{O}+{\text{O}}_{2}$$

(26) $$2H_{2}{\text{O}}_{2}\;\overset{\text{US}}{\to }\;2\cdot \text{OH}$$

It is also possible that the H_2_O_2_ scavenges the insitu formed ^·^OH radicals as follows:

(27) H_2_O_2_ + ^·^OH → H_2_O + HO_2_^·^

(28) HO_2_^·^ + HO_2_^·^ → H_2_O_2_ + O_2_

HO_2_^·^ is a poor oxidant (oxidation potential = 1.7 eV) compared to ^·^OH (oxidation potential = 2.8 eV) or H_2_O_2_ (oxidation potential = 1.77 eV). Consequently, higher concentration of H_2_O_2_ (more than the insitu formed) will scavenge the insitu formed ^·^OH (reaction 27). Part of the ^·^OH radicals formed in the system will be used for the degradation of IC and the other part will be interacting with H_2_O_2_ itself and decomposing it. Thus the net quantum of ^·^OH radicals available for degrading the dye is reduced resulting in the inhibition. Absorption of US by H_2_O_2_ and consequent reactions naturally result in decrease in the US available for activation of the catalyst and the substrate which also leads to decrease in the degradation of IC. Similar results are reported in the case of sono, photo and sonophotocatalytic degradation of organic pollutants in water [8, 27].

It is possible that externally added or insitu formed H_2_O_2_ will utilize at least a few active sites on MnO_2_ for adsorption and/or decomposition thereby reducing the number of sites available for IC. Measurement of the adsorption of IC (60 mg/L) on MnO_2_ (20 mg/L) at various concentrations of H_2_O_2_ (0–80 mg/L) under the sonocatalytic conditions used here, shows that the adsorption of IC is practically unaffected. This confirms that the competitive adsorption of H_2_O_2_ and consequent denial of surface sites for IC on MnO_2_ is not the major cause of the inhibition. It is also observed that the net concentration of H_2_O_2_ present in IC/MnO_2_/US reaction systems with different concentrations of IC, immediately after decolorization, is more or less the same. This steady concentration indicates that the H_2_O_2_ formed insitu is getting decomposed simultaneously. On continued US irradiation, the H_2_O_2_ in the system undergoes oscillation in its concentration. This is consistent with the results reported earlier [5].

**Fig. 10:**
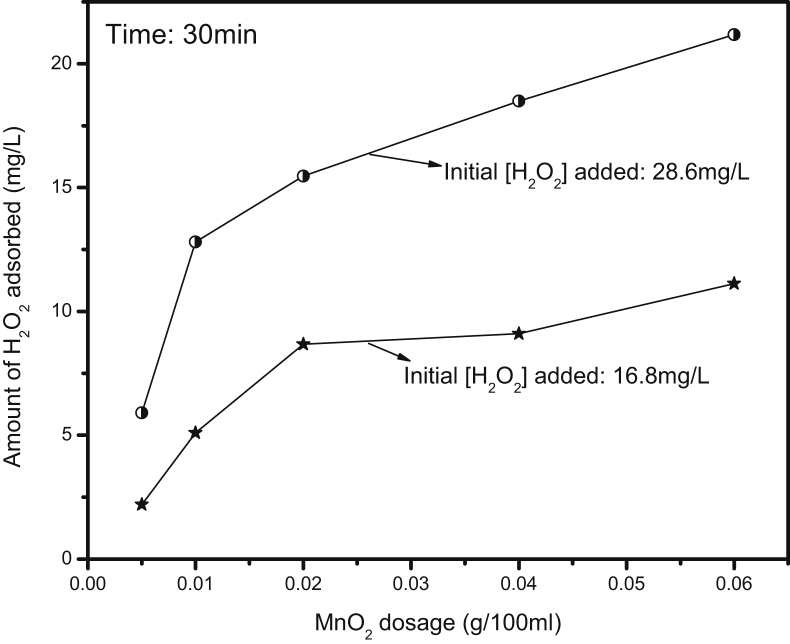
Effect of MnO_2_ dosage on the adsorption/decomposition of H_2_O_2_.

In the absence of IC, the adsorption of H_2_O_2_ increases with increase in dosage of MnO_2_ upto an optimum and stabilizes thereafter (Fig. 10). The same trend is followed when the concentration of H_2_O_2_ is almost doubled (16.5–29 mg/L). The data shows that the adsorption of H_2_O_2_ on MnO_2_ is directly dependent on the concentration of the former and the dosage of the latter. Once the adsorption of H_2_O_2_ is optimum, at least a part of it gets decomposed/desorbed resulting in adsorption-desorption equilibrium. However, in the presence of IC which gets better adsorbed, the adsorption of H_2_O_2_ is not significant and the inhibition is mainly due to other factors as explained earlier.

#### 3.8.2 Effect of persulphate (PS)

**Fig. 11:**
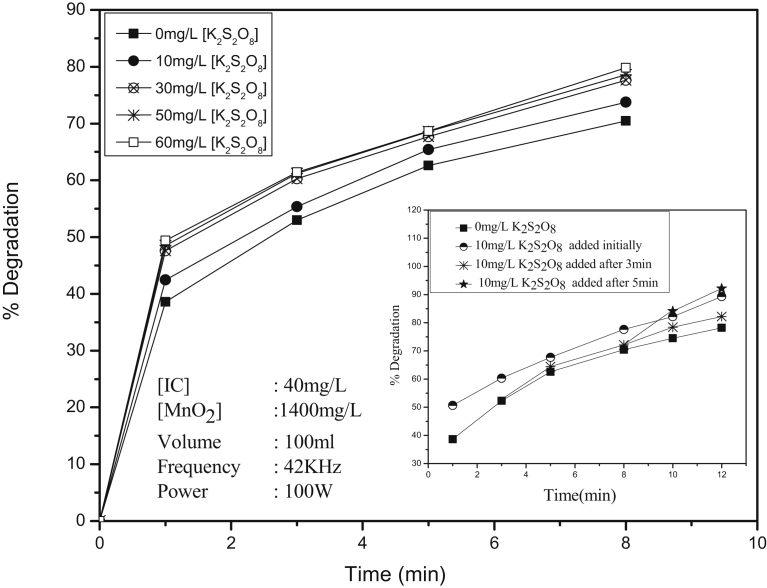
Effect of K_2_S_2_O_8_ on the Sonocatalytic degradation of IC over MnO_2_. [Inset]: Effect of initial and in between addition of K_2_S_2_O_8_ on the degradation of IC.

In the context of inhibition of the sonocatalytic degradation by H_2_O_2_, another oxidant S_2_O_8_^2−^ (E^0^ = 2.1 V) is investigated as a potential enhancer. Persulphate has specific advantages such as high solubility and stability at ambient temperature. Further, the SO_4_^2−^ ions, which are the major products of PS reduction are relatively harmless and considered environment-friendly. PS as such does not cause any degradation of IC. The effect of PS on the reactions is shown in Fig. 11.

The degradation is enhanced moderately in presence of PS in the range of 10–50 mg/L. Thereafter the degradation is stabilized with no enhancing effect by further increase of PS. The enhancement is further confirmed by in-between addition of PS to the degradation reaction in progress (Fig. 11 inset) which shows marginal increase from the point of addition. Since there is no negative effect in the presence of excess of PS, any unused oxidant remaining in the system can be used for the treatment of fresh input of IC pollutant which is important from the angle of commercial application of the process.

Combination of the two oxidants H_2_O_2_ and PS showed that the effect on the degradation is the average of the effects of both.

The enhancement of AOP degradation of organics by PS is explained based on the formation of highly reactive SO_4_^−·^ radical anions insitu. Since the enhancement is only moderate, it may be inferred that the formation of SO_4_^−·^ under US is not as significant as in the case of other radiations such as UV light or microwave. The reactions leading to the formation of SO_4_^−·^ radicals and subsequent interactions with the substrate IC are presented in Eqs. (29), (30), (31), (32), (33), (34), (35), (36), (37), and (38) [74].

(29) S_2_O_8_^2−^ → 2SO_4_^−·^

(30) SO_4_^−·^ + RH → R^·^ + HSO_4_^−^ RH = IC

(31) SO_4_^−·^ + H_2_O → SO_4_^2−^ + H^+^ + ^·^OH

(32) S_2_O_8_^2−^ + RH → SO_4_^−·^ + HSO_4_^−^ + R^·^

(33) ^·^OH + (RH, R^·^) → Intermediates……→ products

(34) SO_4_^−·^ + (R^·^, ^·^OH) → Chain termination

(35) 2SO_4_^−·^ → S_2_O_8_^2−^ Chain termination

(36) 2 ^·^OH → H_2_O_2_ Chain termination

(37) 2R^·^ → RR Chain termination/Further degradation

(38) SO_4_^−·^ + S_2_O_8_^2−^ → SO_4_^2−^ + S_2_O_8_^−·^

Various ROS such as H_2_O_2_, HO_2_^·^, ^·^OH etc. and SO_4_^−·^ formed during the irradiation interact with IC on the surface of the catalyst as well as in the bulk, leading to its degradation into various intermediates and eventual mineralization. However, the degradation is not increasing with increase in concentration of PS, after the initial enhancement probably because the concentration of the ROS formed is not much. Excess of SO_4_^−·^ radicals formed insitu at higher concentration of PS may also be getting deactivated as in reactions (35) and (38). This leads to stabilization in the rate of degradation at higher concentration of PS.

### 3.9 Effect of anions/salts on the degradation

**Fig. 12:**
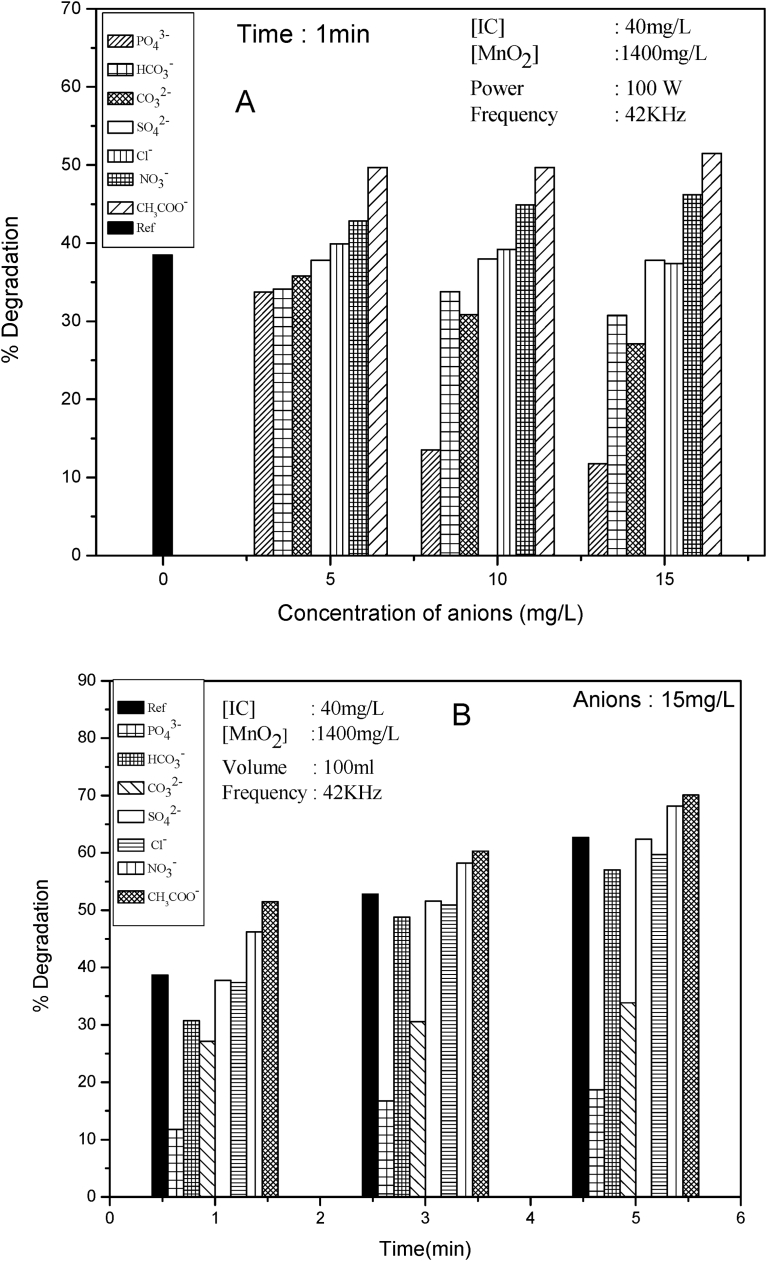
A: Effect of anions at various concentrations on the sonocatalytic degradation of IC, B: Effect of anions at varying reaction times on the sonocatalytic degradation of IC.

Anions are often present in natural water systems and hence the study of the effect of anions on the sonocatalytic degradation of pollutants is important, especially in the context of commercial application of the process. Reports are available on the enhancing as well as inhibiting effect of anions on the sonocatalytic degradation of water pollutants [75]. In the present study the effect of PO_4_^3−^, HCO_3_^−^, CO_3_^2−^, NO_3_^−^, SO_4_^2−^, CH_3_COO^−^ and Cl^−^ on the degradation of IC is investigated under the optimized conditions of other parameters. The common cation in all cases was Na^+^. The anion PO_4_^3−^ inhibits sonocatalytic degradation of IC strongly, the inhibition increasing with increase in concentration of PO_4_^3−^ (Fig. 12A). HCO_3_^−^ and CO_3_^2−^ are mild to moderate inhibitors at all concentrations. SO_4_^2−^ and Cl^−^ have no effect while CH_3_COO^−^ and NO_3_^−^ enhance the degradation moderately. However, as the reaction proceeds and the net concentration of IC decreases, the degree of enhancement decreases, though the trend of inhibition remains practically unchanged at various reaction times within the limits of experimental error (Fig. 12B).

Since the concentration of the salt and the reaction time are known to influence the anion effect significantly [75], detailed investigation on the effect of these two parameters is made and the results are summarized in Table 4.

**Table 4:** Percentage enhancement/inhibition of sonocatalytic degradation of IC in presence of various anions at different concentrations and reaction times.

[MnO_2_]: 100 mg/L, [IC]: 40 mg/L					
Anion	Conc. of anion (mg/L)	% Change at various reaction times^a^			
		1 min	3 min	5 min	8 min
CH_3_COO^−^	2	+16.0	+7.0	+3.7	+2.3
	5	+28.4	+9.7	+7.8	+3.0
	10	+28.4	+11.0	+11.3	+4.7
	15	+33.1	+14.2	+11.8	+5.4
NO_3_^−^	2	+8.0	+5.5	+3.5	+3.0
	5	+15.5	∼0	∼0	∼0
	10	+11.1	+4.5	∼0	∼0
	15	+8.8	+4.6	∼0	∼0
Cl^−^	2	∼0	∼0	∼0	∼0
	5	∼0	∼0	∼0	∼0
	10	∼0	∼0	∼0	∼0
	15	∼0	∼0	∼0	∼0
SO_4_^2−^	2	∼0	∼0	∼0	∼0
	5	∼0	∼0	∼0	∼0
	10	∼0	∼0	∼0	∼0
	15	∼0	∼0	∼0	∼0
CO_3_^2−^	2	∼0	∼0	∼0	∼0
	5	−7.2	−16.2	−15.8	−14.6
	10	−20.2	−27.8	−27.1	−25.7
	15	−29.8	−42.3	−46.0	−41.8
HCO_3_^−^	2	∼0	∼0	∼0	∼0
	5	−8.7	−5.7	−3.9	−5.1
	10	−10.5	−7.0	−6.0	−8.8
	15	−15.2	−8.6	−8.5	−12.6
PO_4_^3−^	2	−9.5	−15.6	−14.2	−12.1
	5	−12.2	−20.7	−20.8	−20.8
	10	−64.6	−67.7	−67.7	−65.2
	15	−69.0	−68.6	−70.1	−70.1

a Negative (−) sign = inhibition, Positive (+) sign = enhancement, ‘0’ = No effect.

The results illustrate that the effect of anions/salts on the sonocatalytic degradation of IC in presence of MnO_2_ is complex and does not follow any consistent pattern with respect to concentration (of the anion) and/or the time of reaction. While inhibitors remain inhibitors always, ‘enhancement’ becomes ‘no effect’ depending on the reaction conditions. Hence the effect of each anion has to be analysed individually in the context of the reaction system, conditions and the composition. The effect, whether inhibition or enhancement, is only modest, except in the case of clear inhibitors (PO_4_^3−^ and CO_3_^2−^) and enhancers (CH_3_COO^−^ and NO_3_^−^) probably because of the microstreaming effect of US on the catalyst particles which result in continuous cleaning of the surface.

The inhibition is often explained based on the preferential adsorption of anions on the catalyst surface and consequent reduction in the availability of surface sites for the generation of ROS and the adsorption of the substrate. In this respect, the adsorption of IC on MnO_2_ in presence of the anions is measured and the results are given in Table 5.

**Table 5:** Effect of anions on the adsorption of IC on MnO_2_.

[IC]: 40 mg/L [MnO_2_]: 1400 mg [Anion]: 10 mg/L	
Anion	Adsorption (%)
Nil (Reference)	32.2
PO_4_^3−^	14.3
CO_3_^2−^	19.4
HCO_3_^−^	24.3
SO_4_^2−^	30.5
Cl^−^	33.5
CH_3_COO^−^	29.5
NO_3_^−^	34.7

The adsorption of IC is inhibited in the order PO_4_^3−^ > CO_3_^2−^ > HCO_3_^−^. Other ions do not affect the adsorption significantly. Comparing with the effect of anions on the degradation of IC, it may be inferred that the inhibition in the degradation can be at least partially attributed to the decrease in the adsorption of the dye. The enhancement in presence of CH_3_COO^−^ and NO_3_^−^ has to be explained based on other factors.

Another possible reason for the inhibition of the degradation may be layer formation by the anions on the catalyst surface. The efficiency of layer formation depends on the solubility of the salts [76], i.e., the more the solubility, the less the layer formation. The solubility of the salts (in mg/g of water at 20 °C) tested here is decreasing in the order;

(39) NaNO_3_ (94.9) > CH_3_COONa (54.6) > Na_2_SO_4_ (40.8) ≥ Na_2_CO_3_ (39.7) > NaCl (36.1) > Na_3_PO_4_ (16.3) > NaHCO_3_ (11.1)

Correspondingly, the order of layer formation must be as follows:

(40) HCO_3_^−^ > PO_4_^3−^ > Cl^−^ > CO_3_^2−^ > SO_4_^2−^ > CH_3_COO^−^ > NO_3_^−^

If the layer formation is at least one of the major causes of inhibition, the degradation should be inhibited in the same order as in (40). This trend is more or less followed at least qualitatively in the current instance. This can partially explain the maximum inhibition in the case of PO_4_^3−^, HCO_3_^−^ and CO_3_^2−^ and the least (where it is even enhancement) in the case of NO_3_^−^ and CH_3_COO^−^. This also confirms that the surface does play an important role in the sonocatalytic degradation of IC, even though the reactions taking place in the bulk are also significant.

Another factor which can influence the effect of anions is their ability to function as ^·^OH radical scavengers. The scavenging rate constants of ^·^OH by some of the anions are summarized in Table 6 [77, 78].

**Table 6:** Scavenging rate constants of ^·^OH by various anions.

Anion	Scavenging rate constants (mol^−1^ s^−1^)
PO_4_^3−^	2 × 10^4^
CO_3_^2−^	3.9 × 10^8^
HCO_3_^−^	8.5 × 10^6^
SO_4_^2−^	1 × 10^10^
Cl^−^	4.3 × 10^9^
CH_3_COO^−^	7.0 × 10^7^
NO_3_^−^	1.4 × 10^8^

The table shows that the inhibition by respective anions cannot be strictly correlated with the scavenging rate constants. Hence it may be inferred that the preferential adsorption of the anion and surface layer formation and consequent impact on the activation of the catalyst surface as well as generation of ROS are mainly responsible for the inhibition.

Since the degradation proceeds moderately well even in the presence of the inhibiting factors as above, there may be other factors which can compensate for them at least partially and assist the degradation. This is more evident in the enhancement of the degradation of IC, in the presence of the two anions CH_3_COO^−^ and NO_3_^−^.

Scavenging of the ^·^OH by anions would yield respective radical anion species [77].

(41) ^·^OH + HCO_3_^−^ → H_2_O + CO_3_^·−^

(42) ^·^OH + CO_3_^2−^ → OH^−^ + CO_3_^·−^

(43) ^·^OH + SO_4_^2−^ → OH^−^ + SO_4_^·−^

(44) ^·^OH + Cl^−^ → OH^−^ + Cl^·^

(45) ^·^OH + NO_3_^−^ → OH^−^ + NO_3_^·^

(46) ^·^OH + CH_3_COO^−^ → OH^−^ + CH_3_COO^·^

(47) CH_3_COO^·^ → CH_3_^·^ + CO_2_

These radical anions are also good oxidants, though the efficiency is less compared to the ^·^OH radicals. They undergo radical-radical recombination and deactivation very slowly. Hence these radical anions are more readily available for longer time than ^·^OH to interact with the substrate and effect degradation. Thus the relatively lower reactivity is partially compensated by the better availability for reaction with the substrate. The ^·^OH radicals would undergo recombination with other ^·^OH radicals and get deactivated or interact with the substrate depending on the availability and proximity. The degradation of the substrate is enhanced when the rate of reactive radical anion formation by interaction between added anions and ^·^OH is faster than the interaction between substrate and ^·^OH. However, both the rates would be much lower than the recombination and consequent deactivation of ^·^OH. In the case of ‘no effect’ the competing factors leading to inhibition and enhancement (as discussed above) may be suitably balanced.

The interaction between the ^·^OH radicals and the anions forming the reactive radical anion takes place at the air-water interface of the cavitation bubbles where polarizable anions can get accumulated. These radical anions diffuse into the solution bulk and react with the substrate. The concentration of the ^·^OH radicals in the bulk is less and hence their recombination is slower. Consequently, the ^·^OH will interact with the more abundant substrate molecules and anions, both leading to enhanced degradation. Hence, indirectly, the anions are shielding the ^·^OH radicals from unproductive recombination by transforming them into radical anions. Towards the later stages of reaction when the degradation of the substrate has progressed substantially, its concentration in the system is less. Hence the radical anion X^−·^ which has only substrate as the sink, cannot sustain the enhanced degradation rate. From that point onwards, the anion-caused enhancement slows down gradually and they may even become inhibitors eventually. This can explain the slowdown or stabilization of the ‘enhancement’ or even its transition to ‘inhibition’ with concentration of the anion and time of reaction.

Another possibility for the anion effect is the variation in the pH, if any, in presence of the anions. However, experiments showed that except in the case of CO_3_^2−^and HCO_3_^·−^, the pH is practically unaffected by other anions. Even in the case of these two anions also pH is increasing only marginally, i.e. from 5.4 in the absence of any anion to 7.1 and 7.8 in the case of HCO_3_^·−^ and CO_3_^2−^ respectively. The effect of pH on the degradation of IC shows that the degradation is more or less the same at pH 5.4, 7.1 or 7.8. Hence variation in pH is not the cause for anion effect in this instance.

Even those anions which are getting competitively better adsorbed on the surface need not remain there for long under sonolytic conditions due to the phenomena of microstreaming and microbubbles eruption [23]. This type of surface cleaning can contribute to sustained long term activity of the catalyst. Since the inhibition is not significant, except in the presence of PO_4_^3−^, HCO_3_^−^ and CO_3_^2−^ which are proven to be strongly adsorbed and hence function as inhibitors, the surface cleaning may indeed be happening in sonocatalysis. This is further evident from the observation that the inhibition of the degradation of IC is not linearly increasing with increase in the concentration of the anion and the degree of inhibition is even decreasing at higher concentration (of the anion).

Accumulation of various reaction intermediates with reaction time which may get more strongly bound to the surface due to structural characteristics can challenge the efficiency of microstreaming. Diminishing concentration of the substrate, competition from the intermediates formed insitu and the anions for the reactive free radicals, relative concentration of the reactive radical anions, reduced penetration of US energy into the system at higher concentration of anions etc. will influence the degree of inhibition. Depending on the relative contributions of various factors, the net effect can be ‘inhibition’, ‘no effect’ or even ‘enhancement’.

Anions increase the ionic strength of the solution leading to ‘salting out’ of the pollutant into the cavitation bubbles where gas phase pyrolysis could take place under sonolysis. This can assist the degradation in certain cases leading to enhancement. Presence of salts can also modify the partition coefficient and hence the distribution of aqueous and organic phases. Solid particles (such as MnO_2_ in the present case) can facilitate better propagation of US in the reaction medium and create instabilities in the system. This will result in enhanced generation of more cavitation bubbles and better emission of light (sonoluminescence) through the reactor [18]. This can lead to photocatalysis and sonophotocatalysis depending on the intensity of light. Suspended solid particles may also provide additional nucleation sites and facilitate surface cavitation due to surface roughness [60, 62]. The formation of crevices and the obstruction by the particles break up the spherical symmetry of the large sized cavitation bubble into many little ones. Increase in number of cavitation bubbles will increase the pyrolysis of water, generate more free radicals and enhance the degradation of IC. The adsorbed anions may also enhance the nucleation capability of the surface which reaches an optimum at certain concentrations. These factors also contribute to increase in the concentration of pollutant at the gas-liquid interface and the cavity implosion sites. This will result in more effective interaction of the substrate with the reactive free radicals leading to enhanced degradation rates. Presence of salt can also decrease the vapor pressure and increase the hydrophilicity as well as surface tension of the aqueous phase resulting in more violent collapse of the cavities and increased degradation. Surface tension can also affect the nucleation process and the cavitational threshold. Thus multitude of complimenting and contradicting factors are operating in presence of anions under sonolysis making the ‘anion effect’ complex, inconsistent and unpredictable.

### 3.10 Identification of reaction intermediates

**Fig. 13:**
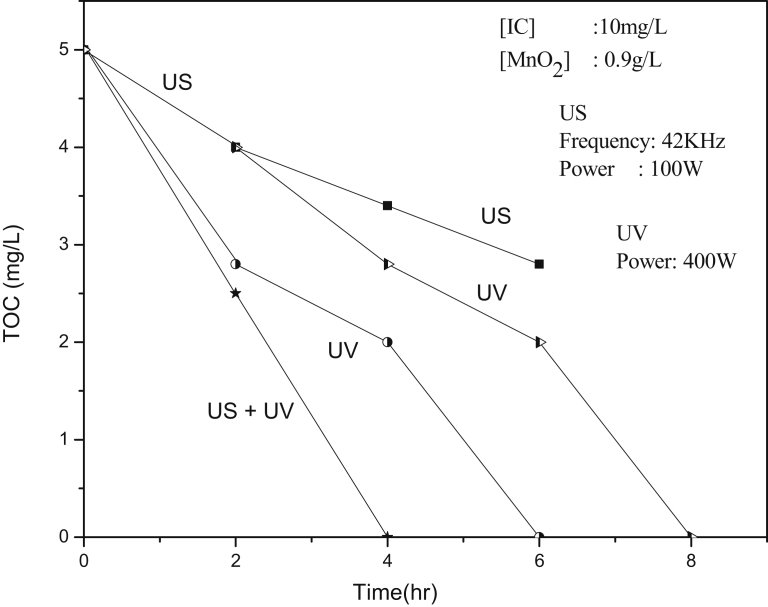
TOC of MnO_2_/IC system under various conditions of activation.

**Fig. 14:**
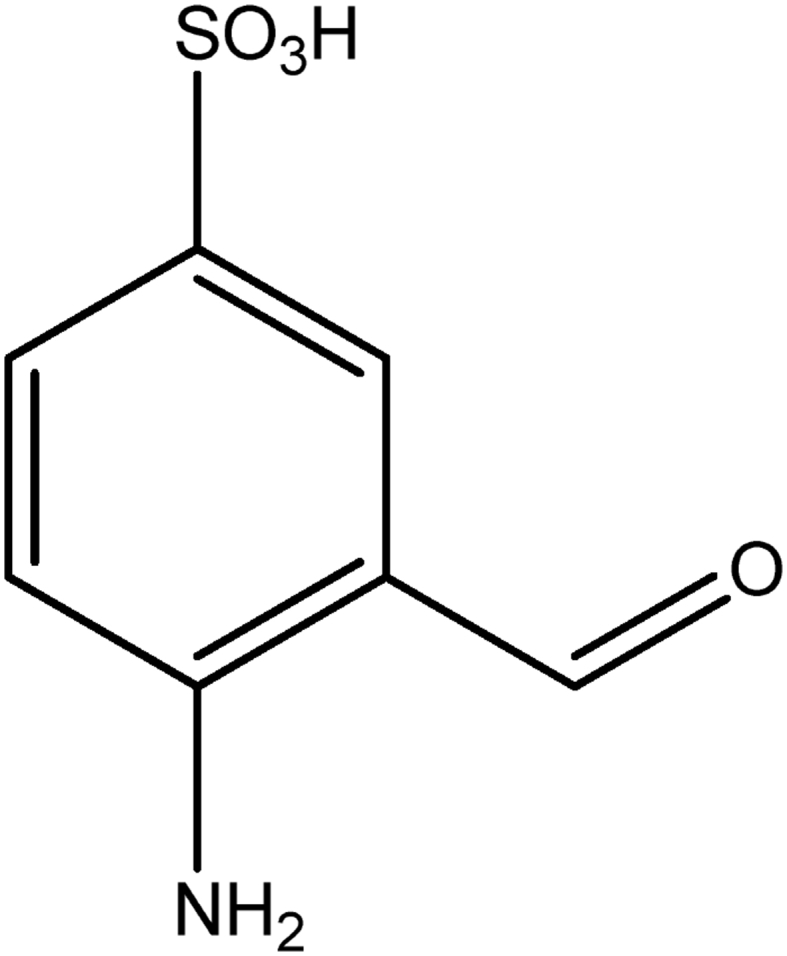
Structure of intermediate 1.

**Fig. 15:**
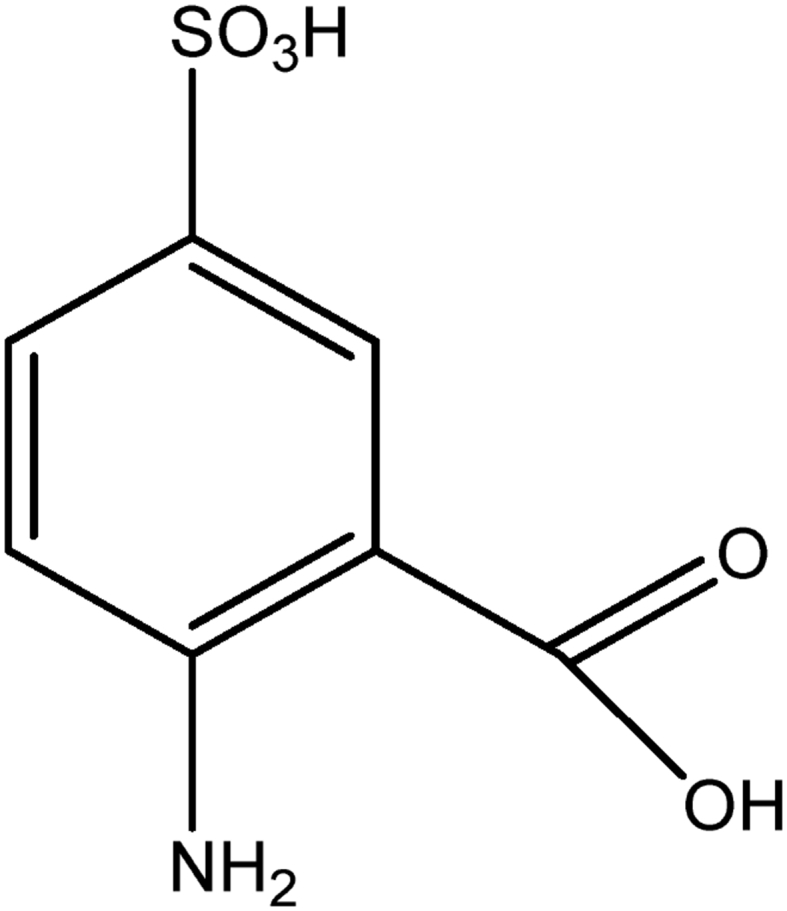
Structure of intermediate 2.

**Fig. 16:**
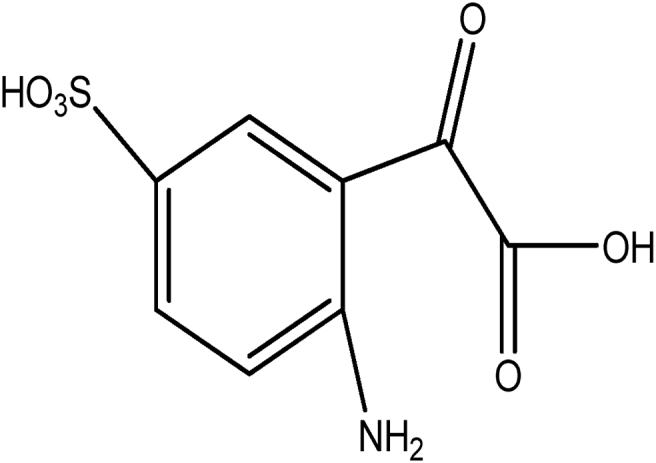
Structure of intermediate 3.

**Fig. 17:**
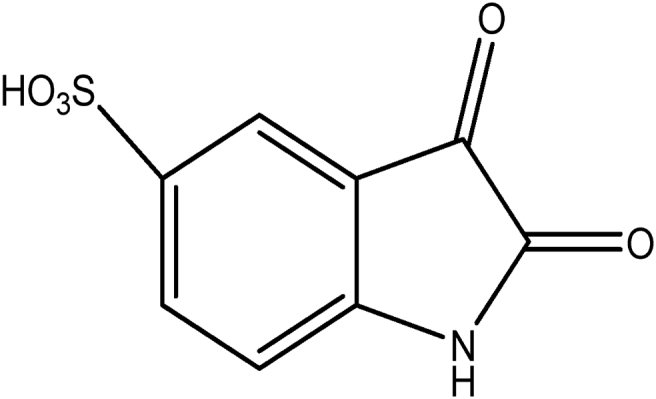
Structure of intermediate 4.

**Fig. 18:**
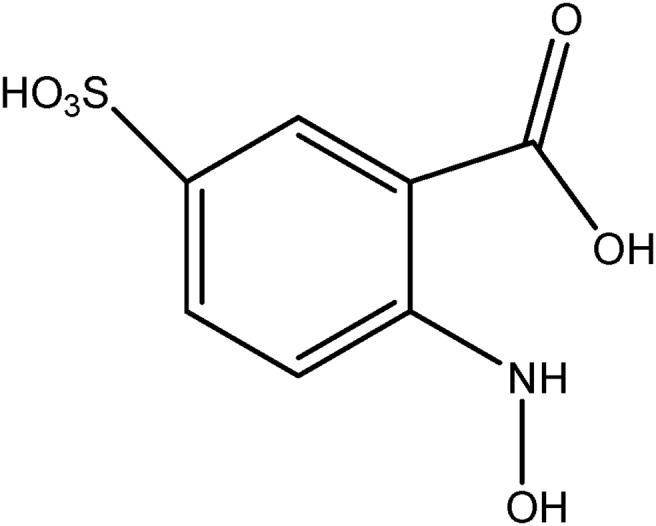
Structure of intermediate 5.

The mineralization of IC by MnO_2_ mediated sonocatalysis is verified by total organic carbon (TOC) measurements. The results are presented in Fig. 13. The TOC of a representative reaction system (IC = 10 mg/L, MnO_2_ = 0.09 g/L) before US irradiation was 5 mg/L. Immediately after decolorization (5 minutes) it becomes 3.5 mg/L. Further US irradiation reduces the TOC very slowly indicating that US irradiation alone is not powerful enough to effect rapid mineralization of the intermediate products. The reaction intermediates present in the system at this stage are identified by LC/MS (Figs. 14, 15, 16, 17, and 18) and are listed in Table 7.

**Table 7:** Major intermediates formed during the sonocatalytic degradation of IC.

Sl No.	m/z	Structure proposed
1	200	Fig. 14
2	217	Fig. 15
3	245	Fig. 16
4	227	Fig. 17
5	233	Fig. 18

Incidentally, the presence of these intermediates provides further evidence to show that the removal of IC in presence of MnO_2_ is not due to simple adsorption. The intermediates can be formed only by degradation of IC. If the decolorization was due to simple adsorption, when the solution was fully decolorized, the entire dye (organic carbon) from the solution would have been removed and the COD/TOC of the supernatant liquid would be ‘nil’. However, in the case of US/MnO_2_/IC, even after many hours of sonication of the decolorized solution, the TOC is remaining steady at ∼ 40% of the original value showing the presence of recalcitrant colorless intermediates in the solution. This reconfirms that the removal of IC from water and its decolorization in presence of MnO_2_/US is not by simple adsorption, but by degradation to form colorless stable intermediates.

If the IC is simply adsorbed on MnO_2_ and not degraded, it must be possible to desorb at least a part of it by re-suspending the used catalyst in water and subjecting it to microwave (MW) irradiation or sonication. However, even after long time (>2 hr) of MW or ultrasound irradiation of the suspension, the water remains colorless. Analysis of this water does not show the presence of IC. This again reconfirms that the decolorization of the dye is due to degradation and not simple adsorption.

The possibility of using photolysis/photocatalysis to drive the sonocatalytic degradation of IC upto complete mineralization is tested by subjecting the decolorized reaction suspension to UV irradiation and determining the TOC at different intervals. The results presented in Fig. 12 show that sonocatalysis followed by photocatalysis leads to complete mineralization (TOC = 0) in 8 hr. Simple UV photocatalysis of the identical system gets mineralized in 6 hr. However, simultaneous sono and photocaytalysis (sonophotocatalysis) was the most efficient with the mineralization completed in 4 hr. This is consistent with the synergy reported in the case of sonophotocatalysis [47, 66]. Hence the efficiency of different AOPs for the mineralization of IC under otherwise identical condition is: sonophotocatalysis (simultaneous) > photocatalysis > (sonocatalysis + photocatalysis in sequence) > > sonocatalysis. However, among these AOPs, sonocatalysis is the most efficient for the decolorization of IC.

### 3.11 General mechanism

Acoustic cavitation produces highly reactive primary radicals such as ^·^OH and H^·^ as in reaction (3). Recombination and a number of other reactions occur on the surface as well as in the bulk as in reactions (48), (49), (50), (51), (52), (53), (54), (55) following this primary radical generation. In the case of sonolysis and sonocatalysis the concentration of ^·^OH radicals at the surface of the cavitation bubble is more compared to that in the solution bulk [18, 55]. The air-water interface of the cavitation bubble also will be rich in OH radicals. All these radicals will not be available exclusively to interact with and degrade IC because of the parallel multiple radical-radical reactions to give H_2_O_2_ and H_2_O. This will also limit the diffusion of radical species to the solution bulk [56]. Various reactions of ^·^OH at the bubble surface and in the bulk may be summarized as follows:

(48) 2^·^OH_(s)_ → H_2_O_2(s)_

(49) ^·^OH_(s)_ + H^·^_(s)_ → H_2_O

(50) H^·^_(s)_+ O_2(s)_ → HO_2_^·^_(s)_

(51) ^·^OH_(s)_ → ^·^OH_(b)_

(52) H^·^_(s)_ → H^·^_(b)_

(53) H^·^_(b)_ + O_2(b)_ → HO_2_^·^_(b)_

(54) 2^·^OH → H_2_O + O^·^

s: bubble surface, b: solution bulk

(55) IC + ROS (H_2_O_2_, ^·^O_2_^−^, HO_2_^·^, OH^·^, h^+^ etc formed under sonocatalysis)) → Intermediates → → → H_2_O + CO_2_ + salts

It is also possible that H_2_O_2_ decomposes under the same conditions as explained earlier.

(56) H_2_O_2_ + US → 2^·^OH

(57) 2H_2_O_2_ + US → 2H_2_O + O_2_

H_2_O_2_ also scavenges the insitu formed ^·^OH radicals as in reaction (27). However, part of H_2_O_2_ will be replenished by reaction (28).

Dissolved oxygen serves as a source of nucleus cavitation [46] which leads to the generation of more reactive species as in reactions (58), (59), (60).

(58) O_2_ → 2O

(59) O_2_ + O → O_3_

(60) O + ^·^O_2_H → ^·^OH + O_2_

The ozone produced as in reaction (59) can decompose as in reaction reaction (61) and the atomic oxygen can produce more ROS (reactions reaction (62), 63).

(61) O_3_ + US → O_2_ + O

(62) O + H_2_O → ^·^OH + ^·^OH

(63) O + H_2_O → H_2_O_2_

The ^·^OH radicals in the bulk are primarily those which escape recombination at the bubble surface [52]. The limited availability of ^·^OH radicals in the bulk will help their efficient utilization by the substrate molecules. The consumption/deactivation of ^·^OH radicals by anions and/or other processes take place mostly at the bubble surface.

The particles of MnO_2_ may get thermally excited and this can lead to the heat-induced catalytic degradation of the IC. The cavitation heat produces holes on the surface of the oxide which can interact with water and produce reactive ^·^OH radicals thereby enhancing the degradation. Similar results are reported in the case of TiO_2_ sonocatalysis as well [79]. In the presence of suspended particles, an endothermic process exists in water upon US irradiation and this will result in the thermal excitation of MnO_2_. However the excitation will occur only to a limited extent since it is difficult for the powder particles to be encapsulated within the cavities.

Since MnO_2_ has been shown to have good photocatalytic activity [30], the sonoluminescence-induced photocatalysis also will be relevant here. US irradiation results in the formation of light of comparatively wider wavelength range (200–500 nm). Light of wavelength <375 nm can excite the semiconductor catalyst leading to the formation of highly active ^·^OH radicals on the surface. MnO_2_ has been proven to be a powerful photocatalyst for the mineralization of IC [30]. Thus the basic mechanism of sonocatalysis can be partly of photocatalysis and possibly of sonophotocatalysis. Absorption of photons by MnO_2_ leads to the formation of electron-hole pairs (reaction 11) which is the first step in photocatalysis. The efficiency of the process depends on the ability to prevent the electron-hole recombination which results in unproductive heat generation (reaction 64).

(64) h^+^ + e^−^ → Heat

The formation of reactive ^·^OH radicals from the electron and hole in presence of O_2_ is shown in reactions (12) and (13). Another reactive free radical HO_2_^·^ also is formed as in reactions (50), (53) and (65).

(65) O_2_^−·^ + H^+^ → HO_2_^·^

These radicals lead to the formation of the oxidant H_2_O_2_ as follows:

(66) O_2_^−·^ + 2 H^+^ + e− → H_2_O_2_

(67) 2HO_2_^·^ → H_2_O_2_ + O_2_

(68) HO_2_^·^ + O_2_^−·^ + H_2_O → H_2_O_2_ + O_2_ + ^·^OH

(69) h^+^_VB_ + OH^−^ → ^·^OH

(70) ^·^OH + ^·^OH → H_2_O_2_

Once sufficient concentration of H_2_O_2_ is reached, its photodecomposition also sets in.

(71) H_2_O_2_ + e^−^ → ^·^OH + OH^−^

(72) $${\text{H}}_{2}{\text{O}}_{2}\overset{\text{hν}}{\to }2^{\cdot }\text{OH}$$

(73) H_2_O_2_ + ^·^O_2_^−^ → 2 ^·^OH + O_2_

**Fig. 19:**
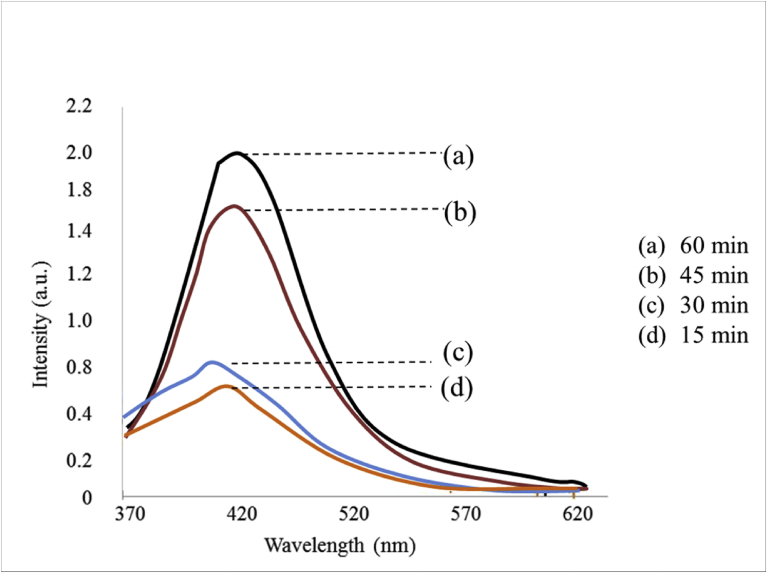
PL spectral changes observed during the US irradiation of MnO_2_ with terephthalic acid, demonstrating the formation of ^·^OH radicals and their increase with time of irradiation.

Thus photocatalysis and possibly mild sonophotocatalysis and consequent degradation of IC are also taking place in the sonocatalytic system. The net concentration of ^·^OH radicals in the system at any point in time depends on the relative rates of their generation and decomposition [5, 75]. The formation of ^·^OH radicals under MnO_2_ sonocatalysis is experimentally verified by the photoluminescence (PL) spectral studies (Section 2.3) as is shown in Fig. 19. The concentration of the radicals increases with time of US irradiation. The oxidation of substrates by ^·^OH radicals can take place both on the surface of the catalyst as well as in the solution bulk. However, current study shows that the sonocatalytic degradation of IC takes place mainly in the bulk. The MnO_2_ after use was subjected to the same physicochemical tests (XRD, SEM, TEM, DRS, FTIR, surface area and particle size) as done before use. The characteristics remain the same except the slight agglomeration of the particles as seen from the SEM analysis.

## 4 Conclusion

MnO_2_ is identified as a highly effective sonocatalyst for the removal of the recalcitrant dye pollutant Indigo Carmine from water. Concentration of the substrate, catalyst loading, pH, reaction volume, frequency of the ultrasound used, availability of O_2_, presence of salts and other contaminants etc. are important parameters that determine the efficiency of the process. The degradation follows variable kinetics which depends on the concentration of the substrate. H_2_O_2_ inhibits the degradation while persulphate which is a very powerful oxidant enhances it moderately. The effect of anions on the degradation varies from ‘inhibition’(PO_4_^3−^, CO_3_^2−^, HCO_3_^−^) to ‘no effect’ (SO_4_^2−^, Cl^−^) or even ‘enhancement’ (NO_3_^−^, CH_3_COO^−^) depending on the reaction conditions and the nature and intensity of interactions. The high affinity of MnO_2_ for O_2_ and its extremely efficient adsorption of H_2_O_2_ and the substrate play key role in the efficiency of the process. Even oxygen from the lattice participates in the reaction whenever there is a deficit of dissolved or adsorbed oxygen. Major transient intermediates formed during the process are identified by LC–MS. Combination of sonocatalysis with UV photolysis (sonophotocatalysis) enhances the efficiency of degradation and mineralization of IC. The observations are discussed and a probable mechanism for the sonocatalytic degradation of the dye is proposed.

## Declarations

### Author contribution statement

Yesodharan Edapoikayil, Suguna Yesodharan, K P Vidya Lekshmi: Conceived and designed the experiments; Analyzed and interpreted the data.

Suguna Yesodharan: Performed the experiments, contributed reagents, materials, analysis tools or data.

K P Vidya Lekshmi: Performed the experiments.

Yesodharan Edapoikayil: Contributed reagents, materials, analysis tools or data.

### Funding statement

This work was supported by the Council of Scientific and Industrial Research (CSIR), Government of India, by way of Senior Research Fellowship to K. P. Vidya Lekshmi.

### Competing interest statement

The authors declare no conflict of interest.

### Additional information

No additional information is available for this paper.
